# Characteristics of interventional clinical trials registered in ClinicalTrials.gov, 2018–2023

**DOI:** 10.1017/cts.2026.10701

**Published:** 2026-03-25

**Authors:** Rebecca D. Sullenger, Robert M. Clare, Ali B. Abbasi, Karen E. Chiswell, Lesley H. Curtis, Bradley G. Hammill, Martin J. Landray, Christopher J. Lindsell, Scott M. Palmer, Sara Bristol Calvert, Adrian F. Hernandez

**Affiliations:** 1 Duke Clinical Research Institute, Duke University School of Medicine, USA; 2 Department of Surgery, University of California San Francisco, USA; 3 Nuffield Department of Population Health, University of Oxford, UK; 4 Clinical Trials Transformation Initiative, USA

**Keywords:** Clinical trials, Clinicaltrials.gov, interventional trials, actionable data, health policy

## Abstract

**Introduction:**

It remains unclear whether the US clinical trial ecosystem is optimized to evaluate medical interventions efficiently. This study characterizes interventional clinical trials in the USA and examines trial progress over five years.

**Methods:**

Using the Aggregate Analysis of ClinicalTrials.gov (AACT) database, we conducted a cross-sectional study of interventional trials initiated in 2023 and a follow-up cohort tracking five-year completion for trials started in 2018 and registered in ClinicalTrials.gov as of 05/01/2024 by sponsor and therapeutic area. Trials with at least one US site were included. Primary outcomes were enrollment, completion status, intervention type, study arm design, and plan to share individual participant data.

**Results:**

Over 7,500 trials met inclusion criteria for each cohort. Most trials started in 2018 (68.4%) and 2023 (62.5%) enrolled fewer than 100 participants. Median enrollment in 2023 was higher for NIH (90) than industry-sponsored (76) trials. Within five years, 60.5% of the 2018 trials were completed, with higher completion among industry (60.9%) than NIH-sponsored (54.3%) trials. In 2023, industry predominantly funded cancer studies and trials testing drug interventions, whereas the NIH prioritized mental health studies and behavioral interventions. More than one-quarter of trials used a single-group design, 72.0% did not plan to share participant-level data, and 72.6% of drug trials were early phase.

**Conclusion:**

Many clinical trials are small, lack a control group, and are incomplete within five years, with differences by sponsor. Given the urgent need to improve health through rigorous evidence generation, there are likely opportunities to improve the US clinical trial ecosystem.

## Introduction

Clinical trials are crucial for assessing the safety and efficacy of medical advances [1], yet are resource and time-intensive [2]. In the USA, bringing a new drug to market often requires investment of hundreds of millions of dollars [3] and more than a decade to progress from phase 1 to regulatory approval.[4] Previous National Institutes of Health (NIH) and National Academy of Medicine (NAM) analyses have recognized inefficiencies in the clinical trial system, which may limit the ability of many trials to advance patient care, including lack of coordination between research groups, inconsistent data standards [5], and trial design characteristics or small enrollment numbers that limit the ability to answer clinically relevant research questions [6]. In response, the NIH and NAM issued a roadmap [5] and a call for a learning health system [7], respectively, to improve clinical trial efficiency by reducing unnecessary delays and resource waste while ensuring patient safety and robust scientific outcomes. An updated understanding of the clinical trial research ecosystem is needed to assess current progress towards these goals and could offer additional insights for more efficient clinical development and evidence generation.

Although small participant sample sizes may be appropriate or necessary in several settings, including in early phase studies [8], and for investigating targeted therapies [9] or rare diseases [10], previous analyses of the US clinical trial landscape have found that many clinical trials are small, use heterogeneous methodologies, and lack randomization. These findings have raised concerns about the collective ability of these trials to inform evidence-based practices and clinical decision-making at the systems level [11–13], catalyzing discussions of the need for significant reforms across the US clinical trial ecosystem [14]. Additionally, US clinical trials often require more sites and enroll fewer patients at higher costs when compared to trials in other countries [15]. A 2012 analysis evaluating interventional trials in three major therapeutic areas (cancer, cardiovascular, and mental health) highlighted possible public health and financial opportunity costs of a clinical evidence generation system dominated by small trials with heterogeneous methods [12]. Nevertheless, funding trends indicate that relatively small trials continue to make up a substantial portion of US studies [16], with one study reporting decreasing median sample size between 2000 and 2019 [13].

Coordinated strategies, including adaptive designs, master protocols, and platform trials [17], can overcome some limitations of small traditional clinical trials and enable agile responses to urgent medical questions [18]. One promising strategy includes master protocols, which are designed with multiple substudies to evaluate one or more interventions for one or more diseases while conserving the resources required to create intervention-specific protocols [19]. Platform studies, which evaluate “multiple medical products for a disease or condition in an ongoing manner,” align with master protocols [19] and have evaluated therapies for various diseases [20–25].

In this article, we characterize the current US interventional clinical trial landscape to inform national policy discussions on optimizing the evidence generation system [14] in the context of recent additions to the NAM’s Learning Health System Series [7], the Clinical Trials Transformation Initiative’s (CTTI’s) Transforming Trials 2030 vision [26], and updated NIH-mandated ClinicalTrials.gov reporting requirements [27]. Using a cohort of interventional trials started in 2018 (allowing us to assess these trials over the subsequent five years) and a cohort of trials started in 2023 (allowing us to assess the characteristics of recently started trials), we explore differences in trial features and disease focus by sponsor, and consider trial characteristics previously described to facilitate the generation of reliable data in an efficient manner, including number of subjects [28], use of a control group, study randomization, and masking [29]. To our knowledge, no studies have characterized the interventional US clinical trial landscape by sponsor or therapeutic area using data within the last five years.

## Methods

### Data source

We used the database for Aggregate Analysis of ClinicalTrials.gov (AACT) [30] to conduct retrospective analyses of clinical research studies registered in ClinicalTrials.gov [31]. AACT is a PostgreSQL relational database, and its schema and data dictionary are detailed elsewhere [32]. A local copy of the AACT database, including data tables as of 5/01/2024, was created as described here [33]. AACT data tables were extracted from the local database [34] and stored as SAS datasets.

Investigators and sponsors register studies as required by applicable laws and policies, including the Food and Drug Administration Amendments Act (FDAAA) [35], the publishing requirements of the International Committee of Medical Journal Editors (ICMJE), or by choice. Information on ClinicalTrials.gov reporting procedures, including required data elements, is detailed elsewhere [36]. The AACT database is updated nightly. All data are based on information provided by the organization responsible for maintaining the study record at ClinicalTrials.gov. Definitions of data elements utilized in this manuscript are available on the ClinicalTrials.gov protocol data element definitions webpage [37]. Except where indicated, variables were used directly from the source data (i.e., no grouping or derivations), as provided by the data submitters.

### Sample selection

We extracted all 493,116 clinical studies registered in ClinicalTrials.gov as of 05/01/2024. Our cross-sectional analysis was limited to interventional trials started in 2023 with at least one US site. ClinicalTrials.gov defines an interventional study as “a type of clinical study in which participants are assigned to groups that receive one or more intervention(s)/treatment(s) (or no intervention) so that researchers can evaluate the effects of the interventions on biomedical or health-related outcomes.” [31] Of note, the terms “clinical study” and “clinical trial” are interchangeable in this work. Our analysis was restricted to trials with at least one US site, defined as a US-based facility with the ability to enroll trial participants, because such trials are more likely to comply with US policies and laws and fully report required information in ClinicalTrials.gov. The same methodology was used to identify interventional studies started in 2018 for the five-year follow-up cohort. We also evaluated the subgroup of phase 3 interventional trials started in 2018. All withdrawn studies, those halted prior to enrollment of the first participant, were excluded. Flow diagrams detailing the sample selection process are in Supplement 1.

### Trial characteristics

Both cohorts were characterized by funding source, based on lead sponsor and collaborator information (eTable 1 in Supplement 1), [industry, NIH, other US federal agencies, other (i.e., individuals, universities, community-based organizations, foundations)]; categorical and median (25th–75th percentile) enrollment; completion status; therapeutic area (cancer, cardiovascular, mental health); intervention type; study phase for studies including a FDA regulated drug/biologic (trials reported as early Phase 1 and Phase 1 were combined, otherwise Phase was recorded exactly as reported by the data submitter); interventional model; number of study sites; site location(s); and plan to share individual participant data. The use of randomization was recorded for studies with more than one arm. Randomized studies were then assessed for masking and data monitoring committee (DMC) appointment, although non-randomized studies may also use DMCs.

A subgroup analysis of phase 3 trials started in 2018 that studied a US FDA-regulated drug or biological product was conducted to compare trials across sponsors with the same intervention type and similar regulatory goals of generating robust enough evidence to perform a risk–benefit analysis of the intervention [38].

We also explored subgroups of cancer, cardiovascular, and mental health trials – conditions that together accounted for over 11,000 disability-adjusted life years lost per 100,000 people in the USA in 2021 [39] – and compared our findings with a 2012 study that considered the same three therapeutic areas [12]. To appropriately assign studies to these three domains, we built on the 2012 methodology [12] by using updated 2024 Medical Subject Heading (MeSH) terms [40] generated by a National Library of Medicine algorithm and user-submitted disease condition terms. Terms that appeared in at least five trials in our final sample were included. All terms were reviewed and annotated for relevance to the therapeutic areas of interest by at least two clinical specialists. A trial was assigned to one or more therapeutic area(s) if annotated with at least one relevant condition term (Supplement 2).

The number of master protocol studies registered in ClinicalTrials.gov was estimated by using the Clinicaltrials.gov “Find Studies” search. The “Other Terms” field was used to search study records for the words: “master protocol” OR “platform trial” OR “basket trial” OR “umbrella trial” OR “multi-arm multi-stage trial.” This terminology is consistent with the FDA Draft Guidance definitions [19] and similar to Park et al. search strategy [41]. Results were filtered to include only the interventional study type. The total number of interventional study records with at least one US site was counted.

### Data analysis

We present categorical characteristics as frequencies and percentages and continuous characteristics as medians and 25^th^–75^th^ percentiles. Summaries were generated using available data. Missing values were excluded. SAS version 9.4 (SAS Institute, Cary, NC) was used for all statistical analyses.

## Results

### General characteristics of trials started in 2023

The general characteristics of the 2023 cohort are presented in Table 1. Of the 493,116 trials downloaded on May 1, 2024, from the AACT database, 7673 met inclusion criteria for the 2023 cohort. Just over half were funded by industry (35.8%) or the NIH (16.9%). Of the therapeutic areas of interest, cancer trials were the most common (23.0%), followed by mental health (19.4%) and cardiovascular studies (10.3%).

**Table 1.** Characteristics of interventional clinical trials started in 2023 and registered in ClinicalTrials.gov by funding source

		Funder type			
	All studies *N* = 7,673	Industry *N* = 2,749	NIH *N* = 1,296	Other US fed *N* = 301	Other *N* = 3,327
Completion status, *n*/*N* (%)					
Not yet recruiting	313 (4.1%)	78 (2.8%)	36 (2.8%)	18 (6.0%)	181 (5.4%)
Recruiting	5646 (73.6%)	1911 (69.5%)	1021 (78.8%)	237 (78.7%)	2477 (74.5%)
Enrolling by invitation	376 (4.9%)	73 (2.7%)	85 (6.6%)	20 (6.6%)	198 (6.0%)
Active, not recruiting	534 (7.0%)	271 (9.9%)	71 (5.5%)	13 (4.3%)	179 (5.4%)
Completed	692 (9.0%)	369 (13.4%)	64 (4.9%)	11 (3.7%)	248 (7.5%)
Suspended	49 (0.6%)	13 (0.5%)	12 (0.9%)	2 (0.7%)	22 (0.7%)
Terminated	62 (0.8%)	34 (1.2%)	6 (0.5%)	0 (0.0%)	22 (0.7%)
Unknown	1 (0.0%)	0 (0.0%)	1 (0.1%)	0 (0.0%)	0 (0.0%)
Therapeutic area^a,b^, *n*/*N* (%)					
Cancer	1768 (23.0%)	828 (30.1%)	309 (23.8%)	25 (8.3%)	606 (18.2%)
Cardiovascular	790 (10.3%)	265 (9.6%)	120 (9.3%)	31 (10.3%)	374 (11.2%)
Mental health	1488 (19.4%)	214 (7.8%)	419 (32.3%)	92 (30.6%)	763 (22.9%)
Intervention types^a^, *n*/*N* (%)					
Drug	3009 (39.2%)	1836 (66.8%)	328 (25.3%)	61 (20.3%)	784 (23.6%)
Device	1,189 (15.5%)	451 (16.4%)	128 (9.9%)	41 (13.6%)	569 (17.1%)
Biological or vaccine	480 (6.3%)	287 (10.4%)	90 (6.9%)	15 (5.0%)	88 (2.6%)
Procedure or surgery	453 (5.9%)	51 (1.9%)	143 (11.0%)	9 (3.0%)	250 (7.5%)
Behavioral	1,978 (25.8%)	60 (2.2%)	638 (49.2%)	136 (45.2%)	1144 (34.4%)
Dietary supplement	242 (3.2%)	62 (2.3%)	27 (2.1%)	13 (4.3%)	140 (4.2%)
Genetic	34 (0.4%)	26 (0.9%)	3 (0.2%)	0 (0.0%)	5 (0.2%)
Radiation	137 (1.8%)	39 (1.4%)	33 (2.5%)	1 (0.3%)	64 (1.9%)
Combination product	80 (1.0%)	45 (1.6%)	7 (0.5%)	1 (0.3%)	27 (0.8%)
Diagnostic test	124 (1.6%)	24 (0.9%)	24 (1.9%)	4 (1.3%)	72 (2.2%)
Other	1,430 (18.6%)	289 (10.5%)	296 (22.8%)	73 (24.3%)	772 (23.2%)
Study phase (for studies with drug interventions^c^), *n*/*N* (%)					
Early Phase 1/Phase 1	1037/3302 (31.4%)	651/2077 (31.3%)	118/340 (34.7%)	17/72 (23.6%)	251/813 (30.9%)
Phase 1/2	355/3302 (10.8%)	246/2077 (11.8%)	27/340 (7.9%)	5/72 (6.9%)	77/813 (9.5%)
Phase 2	1003/3302 (30.4%)	596/2077 (28.7%)	131/340 (38.5%)	31/72 (43.1%)	245/813 (30.1%)
Phase 2/3	82/3302 (2.5%)	52/2077 (2.5%)	9/340 (2.6%)	3/72 (4.2%)	18/813 (2.2%)
Phase 3	517/3302 (15.7%)	438/2077 (21.1%)	27/340 (7.9%)	3/72 (4.2%)	49/813 (6.0%)
Phase 4	308/3302 (9.3%)	94/2077 (4.5%)	28/340 (8.2%)	13/72 (18.1%)	173/813 (21.3%)
Interventional model, *n*/*N* (%)					
Single group	2210 (28.8%)	789 (28.7%)	321 (24.8%)	61 (20.3%)	1039 (31.2%)
Parallel	4091 (53.3%)	1,331 (48.4%)	743 (57.3%)	189 (62.8%)	1828 (54.9%)
Crossover	597 (7.8%)	167 (6.1%)	104 (8.0%)	38 (12.6%)	288 (8.7%)
Sequential	652 (8.5%)	451 (16.4%)	78 (6.0%)	10 (3.3%)	113 (3.4%)
Factorial	123 (1.6%)	11 (0.4%)	50 (3.9%)	3 (1.0%)	59 (1.8%)
Multi-site study, *n*/*N* (%)	2625 (34.2%)	1,730 (62.9%)	337 (26.0%)	83 (27.6%)	475 (14.3%)
Locations of study sites, *n*/*N* (%)					
USA only	6752 (88.0%)	1,920 (69.8%)	1,267 (97.8%)	297 (98.7%)	3,268 (98.2%)
USA and rest of the world	921 (12.0%)	829 (30.2%)	29 (2.2%)	4 (1.3%)	59 (1.8%)
Number of arms, *n*/*N* (%)					
One	2079/7644 (27.2%)	796/2740 (29.1%)	302/1293 (23.4%)	51/301 (16.9%)	930/3310 (28.1%)
Two	3912/7644 (51.2%)	1104/2740 (40.3%)	709/1293 (54.8%)	193/301 (64.1%)	1906/3310 (57.6%)
Three or more	1653/7644 (21.6%)	840/2,740 (30.7%)	282/1293 (21.8%)	57/301 (18.9%)	474/3,310 (14.3%)
Randomized (for studies with >1 arms), *n*/*N* (%)	4728/5565 (85.0%)	1537/1944 (79.1%)	879/991 (88.7%)	226/250 (90.4%)	2086/2380 (87.6%)
Number of parties masked (for randomized studies with >1 arm), *n*/*N* (%)					
No masking	1725/4728 (36.5%)	454/1537 (29.5%)	352/879 (40.0%)	79/226 (35.0%)	840/2086 (40.3%)
1 party masked	978/4728 (20.7%)	124/1537 (8.1%)	239/879 (27.2%)	68/226 (30.1%)	547/2086 (26.2%)
2 or more parties masked	2025/4728 (42.8%)	959/1537 (62.4%)	288/879 (32.8%)	79/226 (35.0%)	699/2086 (33.5%)
Data monitoring committee appointed (for randomized studies with >1 arm), *n*/*N* (%)					
Yes	1867/4728 (39.5%)	706/1537 (45.9%)	482/879 (54.8%)	76/226 (33.6%)	603/2086 (28.9%)
No	2291/4728 (48.5%)	637/1537 (41.4%)	315/879 (35.8%)	139/226 (61.5%)	1200/2086 (57.5%)
Unknown	570/4728 (12.1%)	194/1537 (12.6%)	82/879 (9.3%)	11/226 (4.9%)	283/2086 (13.6%)
Plan to share individual participant data, *n*/*N* (%)					
Yes	1678/6003 (28.0%)	625/2125 (29.4%)	449/1004 (44.7%)	68/267 (25.5%)	536/2607 (20.6%)
No	3969/6003 (66.1%)	1,366/2125 (64.3%)	507/1004 (50.5%)	187/267 (70.0%)	1909/2607 (73.2%)
Undecided	356/6003 (5.9%)	134/2125 (6.3%)	48/1004 (4.8%)	12/267 (4.5%)	162/2607 (6.2%)

a Rows are not mutually exclusive. A study may be counted in more than one row.

b Conditions grouped as in 2012 Califf paper, supplemented with clinical review of new terms occurring in 5 or more trials. Condition groupings are not exhaustive.

c Studies a US FDA-regulated Drug Product: A “US FDA-regulated drug or biological product” is a separate field in ClinicalTrials.gov in which trial investigators are able to “Indicate whether this study includes an intervention subject to US Food and Drug Administration regulation under section 351 of the Public Health Service Act or any of the following sections of the Federal Food, Drug, and Cosmetic Act: 505, 510(k), 515, 520(m), and 522. Select Yes/No.”

Most trials were small, with 63.5% enrolling 100 participants or fewer and 95.7% enrolling fewer than 1001. At the time of data download, 13.7% of trials reported actual, and 86.3% reported anticipated enrollment. Drug studies were the most common (39.2%), followed by behavioral interventions (25.8%). Of the drug interventions, most (72.6%) were earlier-phase trials (i.e., phases 1 through 2). Over half of studies (53.3%) used a parallel assignment model, and more than one in four (28.8%) used a single group design. Approximately one-third (34.2%) of trials enrolled at multiple sites, and fewer than one in seven (12.0%) had sites in both the USA and the rest of the world.

Most trials (66.1%) did not plan to share individual participant data. The majority of studies with more than one arm were randomized (85.0%). Of these, 63.5% used some form of masking, and less than half (39.5%) reported DMC use.

### Characteristics of trials started in 2023 by sponsor

Industry-funded trials were more likely to relate to cancer (30.1%) than were studies funded by the NIH (23.8%). Conversely, industry-funded trials were less likely to relate to mental health (7.8%) than were NIH studies (32.3%). The proportion of cardiovascular studies was similar (9.6% for industry vs. 9.3% for NIH).

**Figure 1.**
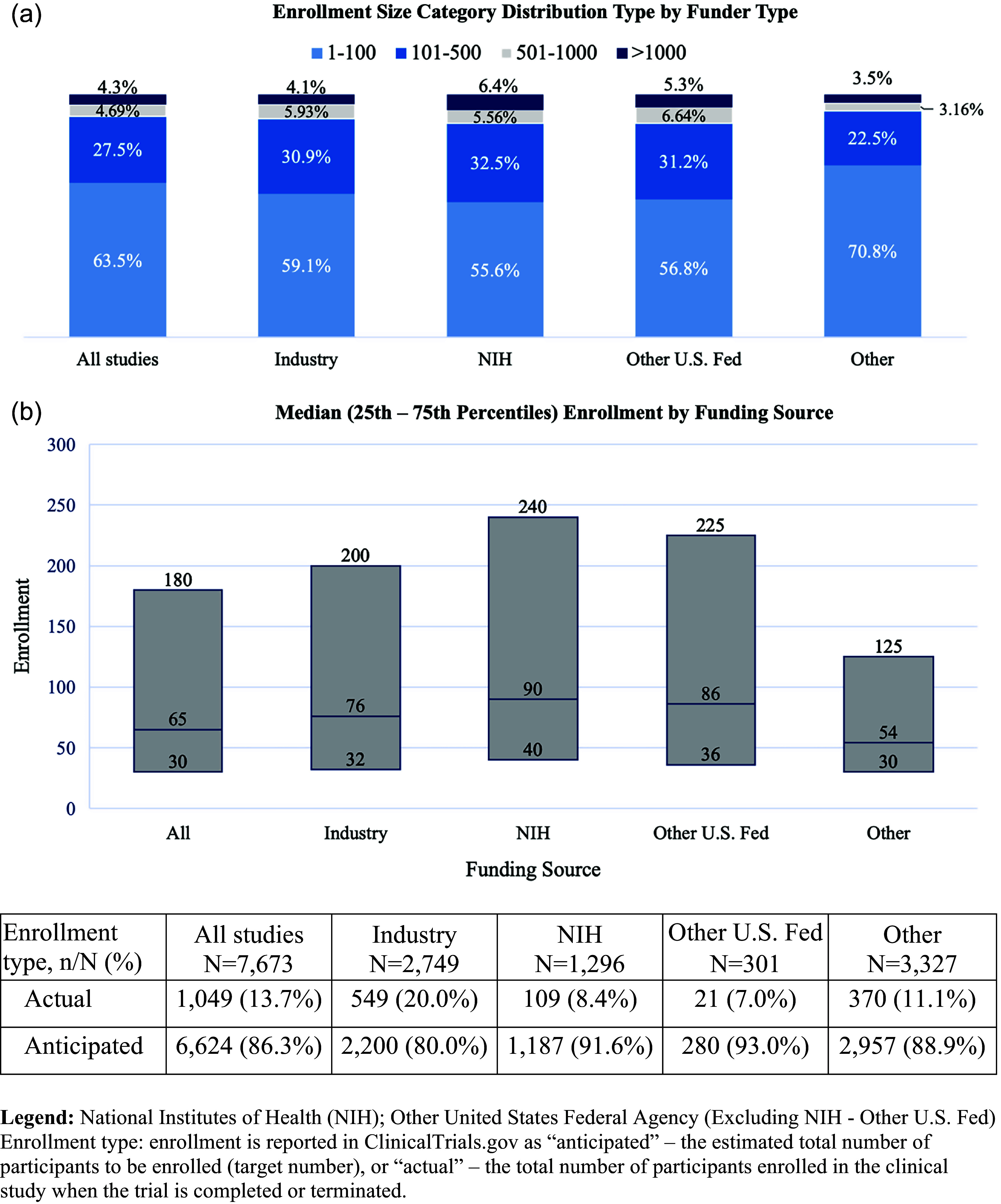
Enrollment for interventional clinical trials started in 2023 and registered in ClinicalTrials.gov by funding source. Legend: National Institutes of Health (NIH); Other United States Federal Agency (Excluding NIH – Other US Fed) Enrollment type: enrollment is reported in ClinicalTrials.gov as “anticipated” – the estimated total number of participants to be enrolled (target number), or “actual” – the total number of participants enrolled in the clinical study when the trial is completed or terminated.

Median enrollment is stratified by funder in Figure 1 and was fewer than 100 individuals across sponsors (76 for industry vs. 90 for NIH vs. 86 for other US federal vs. 54 for other). Most industry-sponsored studies used a drug intervention (66.8%) compared to 25.3% of NIH studies. The NIH was more likely to study behavioral interventions (49.2%) than was industry (2.2%). Of the drug studies, industry-sponsored trials were more likely to be later phase (i.e., phases 3 and 4) than were NIH-funded studies (25.6% vs. 16.1%).

Sponsors also differed in the geographic distribution of sites; 30.2% of industry-funded studies used sites in other parts of the world, whereas less than 5% of studies sponsored by any other group had international sites. The majority of industry-sponsored studies enrolled at multiple sites (62.9%) compared to 26.0% of NIH studies.

Multi-arm NIH studies were more likely than industry studies to use randomization (88.7% vs. 79.1%), whereas industry-sponsored trials were more likely to employ masking (68.5% vs. 60.0% for NIH). NIH trials were more likely to plan to share individual participant data (44.7%) than those funded by industry (29.4%).

### Characteristics of trials started in 2023 by therapeutic area

The characteristics of trials started in 2023 by therapeutic area are presented in eTable 2 in Supplement 1. Mental health trials had the highest median enrollment (25^th^–75^th^ percentile), 75 (40, 200), followed by cardiovascular, 70 (30, 210), and cancer, 60 (30, 150). Most mental health studies used a behavioral intervention (55.2%), in contrast to the majority of drug interventions in cancer studies (61.0%). Later phase trials that investigated an FDA-regulated drug or biologic were more common in cardiovascular (30.8%) and mental health studies (26.4%) than cancer studies (11.1%).

Trial design also differed by therapeutic area. Single group assignment was the most common for cancer trials (41.7%), followed by cardiovascular (29.9%) and mental health trials (23.2%). Of the studies with more than one arm, 63.4% of cancer studies were randomized (vs. 89.0% for cardiovascular vs. 94.4% for mental health). The majority of randomized cancer trials (69.1%) were not masked (vs. 36.2% for cardiovascular vs. 33.9% for mental health). Over half (55.1%) of cancer studies used a DMC (vs. 45.1% for cardiovascular and 40.1% for mental health).

### Differences in trial characteristics by start year

The characteristics of the 2018 trials by sponsor (*n* = 7993) are presented in Table 2. The distribution of primary sponsorship shifted modestly between 2018 and 2023. Industry-sponsored trials decreased from 39.5% in 2018 to 35.8% in 2023, whereas the proportion of NIH-funded studies increased from 14.9% to 16.9%. The relative proportion of mental health studies increased from 2018 (15.0%) to 2023 (19.4%), while the percentage of cancer and cardiovascular trials remained relatively consistent.

**Table 2.** Five-year follow-up characteristics of interventional clinical trials started in 2018 and registered in ClinicalTrials.gov by funding source

		Funder type			
	All studies *N* = 7993	Industry *N* = 3155	NIH *N* = 1187	Other US Fed *N* = 315	Other *N* = 3,336
Enrollment (categorical), *n*/*N* (%)					
1–100	5470 (68.4%)	1959 (62.1%)	790 (66.6%)	191(60.6%)	2530 (75.8%)
101–500	1935 (24.2%)	900 (28.5%)	300 (25.3%)	103(32.7%)	632 (18.9%)
501–1000	342 (4.3%)	194 (6.1%)	56 (4.7%)	7(2.2%)	85 (2.5%)
>1000	246 (3.1%)	102 (3.2%)	41 (3.5%)	14(4.4%)	89 (2.7%)
Enrollment (median)					
Median (Q1, Q3)	50.00 (22.00, 138.00)	60.00 (24.00, 184.00)	56.00 (26.00, 159.00)	72.00 (28.00, 169.00)	42.50 (20.00, 100.00)
Enrollment type, *n*/*N* (%)					
Actual	6545 (81.9%)	2718 (86.1%)	916 (77.2%)	256 (81.3%)	2655 (79.6%)
Anticipated	1448 (18.1%)	437 (13.9%)	271(22.8%)	59 (18.7%)	681 (20.4%)
Completion status, *n*/*N* (%)					
Not yet recruiting	1 (0.0%)	0 (0.0%)	0 (0.0%)	1(0.3%)	0 (0.0%)
Recruiting	665 (8.3%)	157 (5.0%)	167 (14.1%)	24(7.6%)	317 (9.5%)
Enrolling by invitation	60 (0.8%)	12 (0.4%)	6 (0.5%)	2(0.6%)	40 (1.2%)
Active, not recruiting	957 (12.0%)	398 (12.6%)	230 (19.4%)	29(9.2%)	300 (9.0%)
Completed	4832 (60.5%)	1922 (60.9%)	644 (54.3%)	205(65.1%)	2061 (61.8%)
Suspended	58 (0.7%)	13 (0.4%)	11 (0.9%)	0(0.0%)	34 (1.0%)
Terminated	969 (12.1%)	490 (15.5%)	106 (8.9%)	24(7.6%)	349 (10.5%)
Unknown	451 (5.6%)	163 (5.2%)	23 (1.9%)	30(9.5%)	235 (7.0%)
Therapeutic area^a,b^, *n*/*N* (%)					
Cancer	1873 (23.4%)	909 (28.8%)	385 (32.4%)	22(7.0%)	557 (16.7%)
Cardiovascular	787 (9.8%)	311 (9.9%)	93 (7.8%)	25(7.9%)	358 (10.7%)
Mental health	1196 (15.0%)	224 (7.1%)	263 (22.2%)	95(30.2%)	614 (18.4%)
Intervention types^a^, *n*/*N* (%)					
Drug	3570 (44.7%)	2108 (66.8%)	432 (36.4%)	69(21.9%)	961 (28.8%)
Device	1223 (15.3%)	592 (18.8%)	96 (8.1%)	51(16.2%)	484 (14.5%)
Biological or vaccine	625 (7.8%)	364 (11.5%)	167 (14.1%)	16(5.1%)	78 (2.3%)
Procedure or surgery	478 (6.0%)	82 (2.6%)	134 (11.3%)	16(5.1%)	246 (7.4%)
Behavioral	1551 (19.4%)	66 (2.1%)	405 (34.1%)	125(39.7%)	955 (28.6%)
Dietary supplement	261 (3.3%)	56 (1.8%)	29 (2.4%)	7(2.2%)	169 (5.1%)
Genetic	28 (0.4%)	15 (0.5%)	1 (0.1%)	0(0.0%)	12 (0.4%)
Radiation	201 (2.5%)	60 (1.9%)	52 (4.4%)	3(1.0%)	86 (2.6%)
Combination product	70 (0.9%)	41 (1.3%)	3 (0.3%)	2(0.6%)	24 (0.7%)
Diagnostic test	118 (1.5%)	35 (1.1%)	15 (1.3%)	3(1.0%)	65 (1.9%)
Other	1588 (19.9%)	338 (10.7%)	371 (31.3%)	77(24.4%)	802 (24.0%)
Study phase (for studies with drug interventions^c^), *n*/*N* (%)					
Early Phase 1/Phase 1	1174/3924 (29.9%)	679/2342 (29.0%)	185/519 (35.6%)	19/81 (23.5%)	291/982 (29.6%)
Phase 1/2	353/3924 (9.0%)	217/2342 (9.3%)	51/519 (9.8%)	4/81 (4.9%)	81/982 (8.2%)
Phase 2	1195/3924 (30.5%)	704/2342 (30.1%)	204/519 (39.3%)	26/81 (32.1%)	261/982 (26.6%)
Phase 2/3	92/3924 (2.3%)	42/2342 (1.8%)	12/519 (2.3%)	5/81 (6.2%)	33/982 (3.4%)
Phase 3	677/3924 (17.3%)	582/2342 (24.9%)	26/519 (5.0%)	8/81 (9.9%)	61/982 (6.2%)
Phase 4	433/3924 (11.0%)	118/2342 (5.0%)	41/519 (7.9%)	19/81 (23.5%)	255/982 (26.0%)
Interventional model, *n*/*N* (%)					
Single group	2397 (30.0%)	1035 (32.8%)	345 (29.1%)	52(16.5%)	965 (28.9%)
Parallel	4403 (55.1%)	1591 (50.4%)	687 (57.9%)	204(64.8%)	1,921 (57.6%)
Crossover	639 (8.0%)	228 (7.2%)	73 (6.1%)	43(13.7%)	295 (8.8%)
Sequential	441 (5.5%)	290 (9.2%)	58 (4.9%)	8(2.5%)	85 (2.5%)
Factorial	113 (1.4%)	11 (0.3%)	24 (2.0%)	8(2.5%)	70 (2.1%)
Multi-site study, *n*/*N* (%)	3016 (37.7%)	2070 (65.6%)	364 (30.7%)	88(27.9%)	494 (14.8%)
Locations of study sites, *n*/*N* (%)					
USA only	6879 (86.1%)	2,154 (68.3%)	1,137 (95.8%)	312(99.0%)	3276 (98.2%)
USA and rest of the world	1114 (13.9%)	1001 (31.7%)	50 (4.2%)	3(1.0%)	60 (1.8%)
Number of arms, *n*/*N* (%)					
One	2246/7969 (28.2%)	989/3144 (31.5%)	320/1186 (27.0%)	45/315 (14.3%)	892/3324 (26.8%)
Two	3937/7969 (49.4%)	1231/3144 (39.2%)	601/1186 (50.7%)	214/315 (67.9%)	1891/3324 (56.9%)
Three or more	1786/7969 (22.4%)	924/3144 (29.4%)	265/1186 (22.3%)	56/315 (17.8%)	541/3324 (16.3%)
Randomized (for studies with >1 arms), *n*/*N* (%)	4762/5723 (83.2%)	1684/2155 (78.1%)	716/866 (82.7%)	245/270 (90.7%)	2117/2432 (87.0%)
Number of parties masked (for randomized studies with >1 arm), *n*/*N* (%)					
No masking	1679/4762 (35.3%)	464/1684 (27.6%)	281/716 (39.2%)	85/245 (34.7%)	849/2117 (40.1%)
1 party masked	947/4762 (19.9%)	144/1684 (8.6%)	169/716 (23.6%)	79/245 (32.2%)	555/2117 (26.2%)
2 or more parties masked	2136/4762 (44.9%)	1076/1684 (63.9%)	266/716 (37.2%)	81/245 (33.1%)	713/2117 (33.7%)
Data monitoring committee appointed (for randomized studies with >1 arm), *n*/*N* (%)					
Yes	1701/4762 (35.7%)	672/1684 (39.9%)	367/716 (51.3%)	80/245 (32.7%)	582/2117 (27.5%)
No	2578/4762 (54.1%)	836/1684 (49.6%)	286/716 (39.9%)	147/245 (60.0%)	1309/2117 (61.8%)
Unknown	483/4762 (10.1%)	176/1684 (10.5%)	63/716 (8.8%)	18/245 (7.3%)	226/2117 (10.7%)
Plan to share individual participant data, *n*/*N* (%)					
Yes	935/5644 (16.6%)	501/2224 (22.5%)	179/710 (25.2%)	38/270 (14.1%)	217/2440 (8.9%)
No	3999/5644 (70.9%)	1473/2224 (66.2%)	457/710 (64.4%)	211/270 (78.1%)	1858/2440 (76.1%)
Undecided	710/5644 (12.6%)	250/2224 (11.2%)	74/710 (10.4%)	21/270 (7.8%)	365/2440 (15.0%)

a Rows are not mutually exclusive. A study may be counted in more than one row.

b Conditions grouped as in 2012 Califf paper, supplemented with clinical review of new terms occurring in 5 or more trials. Condition groupings are not exhaustive.

c Studies a US FDA-regulated Drug Product. A “US FDA-regulated drug or biological product” is a separate field in ClinicalTrials.gov in which trial investigators are able to “Indicate whether this study includes an intervention subject to US Food and Drug Administration regulation under section 351 of the Public Health Service Act or any of the following sections of the Federal Food, Drug, and Cosmetic Act: 505, 510(k), 515, 520(m), and 522. Select Yes/No.”

**Figure 2.**
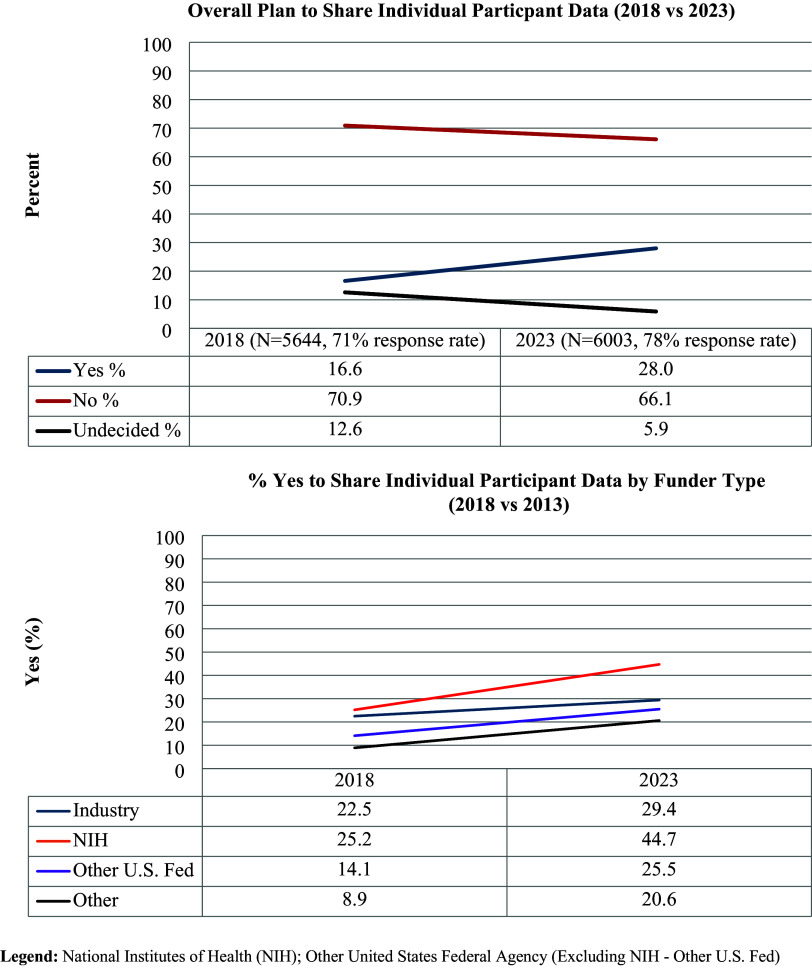
Plan to share individual participant data for interventional trials started in 2018 and 2023 and registered in ClinicalTrials.gov. Legend: National Institutes of Health (NIH); Other United States Federal Agency (Excluding NIH – Other US Fed).

Similarly to 2023, in 2018, of the industry-sponsored studies, 28.8% were cancer studies, and 7.1% were mental health trials. Conversely, the proportion of NIH-funded mental health studies increased (22.2% in 2018 vs. 32.3% in 2023), while the percentage of NIH cancer studies decreased (32.4% in 2018 vs. 23.8% in 2023). The overall proportion of trials that used a drug intervention decreased from 44.7% to 39.2% during the 2 periods, while behavioral health interventions became more common (19.4% in 2018 vs. 25.8% in 2023). The percentage of funders who planned to share individual patient data increased from 16.6% in 2018 to 28.0% in 2023 (Figure 2).

Among the 7,673 trials started in 2023, only 28 (0.4%) met our search criteria for use of a master protocol, which was marginally higher compared to trials started in 2018 (13/7993 (0.2%)).

### Characteristics of phase 3 trials started in 2018

The general characteristics of 2018 phase 3 interventional clinical trials that studied an FDA-regulated drug or biologic (*n* = 677) are presented in eTable 3 in Supplement 1. Industry funded 86% (*n* = 582) of these trials. Cancer trials were the most common (21.0%), and mental health studies were the least common (7.8%). Most trials reported actual enrollment (88.2% vs. 11.8% anticipated). Median (25^th^–75^th^ percentile) enrollment for industry-funded trials was the greatest [358 (140–631)], followed by NIH-sponsored studies [240 (126–690)]. Parallel intervention (81.8%) was the most common arm design, followed by single group assignment (14.5%). In contrast to trials overall, phase 3 trials were more likely to have sites in and outside of the USA (61.0%) than in the USA alone (39.0%).

### Trial completion

**Figure 3.**
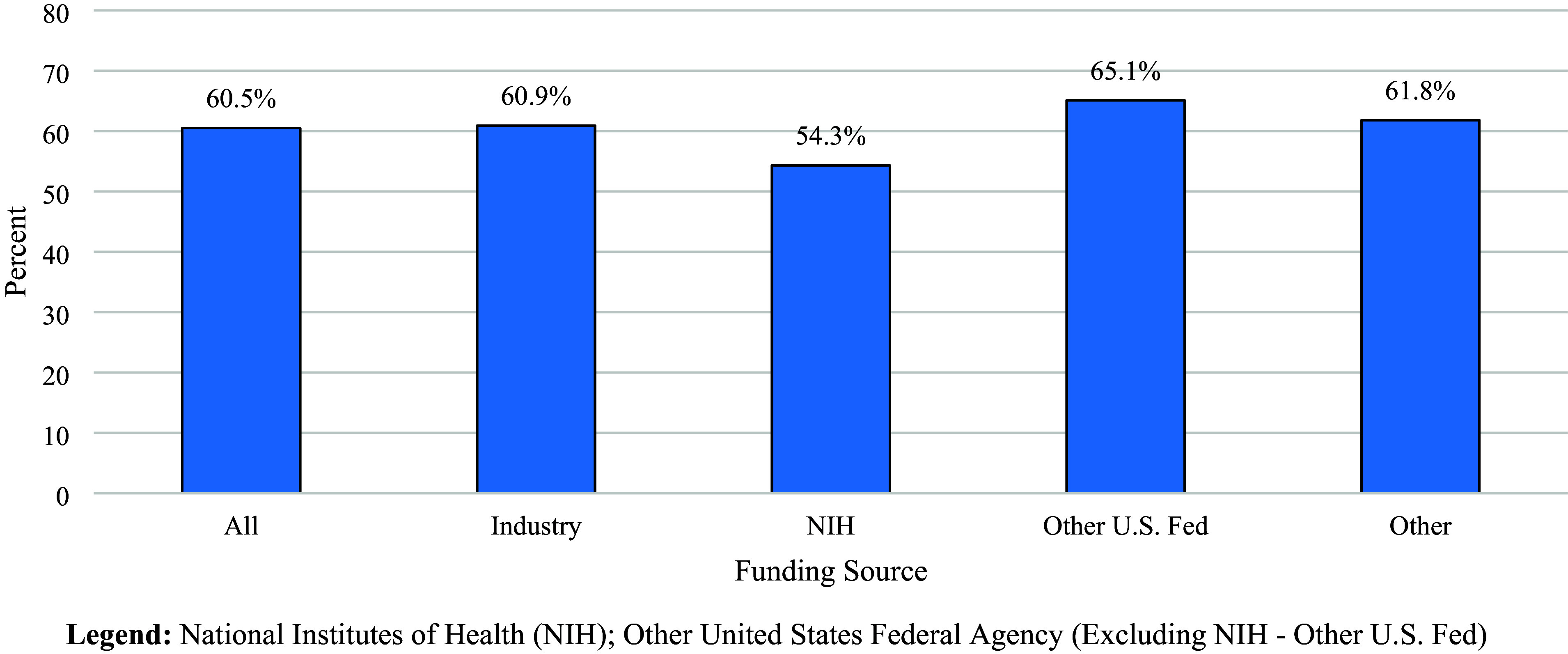
Percent of Interventional clinical trials started in 2018 and completed within five years and registered in ClinicalTrials.gov by funding source. Legend: National Institutes of Health (NIH); Other United States Federal Agency (Excluding NIH – Other US Fed).

The five-year completion rates of the 2018 trials are presented by funding source in Figure 3. Other US federal-sponsored trials were the most likely to be completed (65.1%), followed by other (61.8%), industry (60.9%), and the NIH (54.3%).

## Discussion

A robust, efficient clinical trial system is critical for evaluating the safety and efficacy of rapidly evolving biomedical innovations needed to address urgent public health problems. This analysis of the US interventional clinical trial landscape reveals that many trials remain small, lack a control group, and are incomplete after five years. Although clinical trials should vary based on study objectives, populations, and outcomes, examining study characteristics across a national trial portfolio provides insight into the ability of the current evidence generation system to produce reliable and actionable data.

Trial size and interventional design are key to evaluating a clinical study’s capacity to inform regulatory policy and clinical decision-making [28]. Small and uncontrolled clinical trials are appropriate in several contexts [8], including to study rare diseases [10], or conduct early phase drug evaluations. Additionally, small, targeted cancer therapy studies can produce sufficient data for drug approval [9]. Single-arm studies may also be appropriate in specific ethical contexts, such as in the treatment of patients with advanced cancer [42]. Thus, a more detailed analysis evaluating individual trial indications would likely explain some of our findings.

Nevertheless, conducting numerous small and uncontrolled trials may have important implications for the US evidence generation system. The ability of these trials to detect modest treatment effects or compare similar interventions should be weighed against the direct and opportunity costs for participants. We found that most trials enrolled 100 participants or fewer, and more than a quarter lacked a control group. Even among the 2018 phase 3 trials, 22.7% and 88.7% enrolled 100 and 1,000 or fewer participants, respectively. Additionally, the high proportion of 2018 trials that were not completed within five years raises concerns about the timeliness of data generation.

Despite calls to modernize the clinical trial ecosystem [14], many of our findings mirror those of a prior investigation of interventional clinical trials registered between 2007 and 2010 [12]. In 2023, 63.5% of trials planned to enroll or enrolled 100 or fewer participants, similar to the 62.3% anticipated enrollment for 2007–2010 trials. Likewise, the use of a single group interventional model (31.2% in 2007–2010 vs. 30.0% in 2018 vs. 28.8% in 2023) and the proportion of industry sponsorship (37.2% in 2007–2010 vs. 39.4% in 2018 vs. 35.8% in 2023) were relatively stable over the years. Cancer trials remained the most common of the therapeutic areas, and cardiovascular trials continued to be the least common. Notably, the percentage of mental health trials more than doubled between 2007–2010 (9.0 %) and 2023 (19.4%), possibly reflecting increased investment in mental health care and research [43].

A prior study of completed interventional trials started between 2000 and 2019 found that trial design and time to completion varied by funding source [13]. Our analysis builds on these findings by examining more recent data, applying a uniform 5-year completion cutoff point, and considering completed and uncompleted trials, both of which require significant resources. By additionally assessing trial differences by therapeutic area and restricting our sample to trials with at least one US site, our findings also provide more direct insight into opportunities to strengthen the US clinical trial system.

In addition to characterizing key features of interventional trials, this study provides an update on data sharing practices for clinical trials. Although the ClinicalTrials.gov requirement to include an individual participant data sharing statement was not in effect for trials registered between 2007 and 2010 [44], it was in 2018 and 2023. Most trials did not plan to share individual participant data, regardless of start year or sponsor. Although the percentage of trials that planned to share data increased from 16.6% in 2018 to 28.0% in 2023, these percentages indicate continued hesitancy towards greater data access and transparency despite national interests and ICMJE calls for increased data sharing [45]. NIH-funded trials were the most likely to agree to share individual participant data, possibly influenced by the NIH’s Data Management and Sharing policy [46]. These results suggest the need for stronger data sharing requirements and accountability rules to expand data access.

### Policy strategies

Several policy strategies could improve the US evidence generation system. Leveraging modern, fit-for-purpose trial designs and investing in a more coordinated, research-ready infrastructure with appropriate incentives would likely improve trial efficiency, allowing for an increase in the proportion of larger, more robust trials able to generate conclusive evidence to inform clinical practice [47,48].

Nevertheless, the adoption of master protocols and platform trials remains limited. Despite increased uptake during the COVID-19 public health emergency, only 221 US-based master protocol studies had been registered in ClinicalTrials.gov through 2024 [49]. The NIH, in particular, may be well-positioned to better leverage the benefits of platform trials, as it likely faces fewer concerns than industry regarding pharmaceutical competitiveness [50].

Policies, procedures, and incentives that support a reusable research infrastructure with turn-key processes would complement coordinated trial designs. Recent increases in administrative processes and total cost per trial participant [51] are particularly concerning, and reversing this trend will require policy change. Policy priorities should include streamlining start-up processes, institutional review board approvals, contracting, and enabling scalable technologies and data systems to support greater trial participation. These changes will be especially important to facilitate the rigorous evidence generation urgently needed to improve chronic disease outcomes [52]. Although cardiovascular disease remains the leading cause of death in the USA and its related risk factors are increasing in prevalence [53], we found that only 10% of trials studied cardiovascular disease, suggesting that the current system will need to adjust incentives to better address this major disease burden. Policies that require periodic site and investigator certifications and processes for public reporting of key performance indicators and pay-for-performance results may also improve the clinical trial ecosystem.

### Limitations

This study has important limitations. ClinicalTrials.gov does not include all US clinical trials. Although all “applicable clinical trials” and NIH-funded interventional studies must be registered, and ICMJE policy strongly encourages public registration [54], select trials may be absent from ClinicalTrials.gov. Additionally, our approximation of the number of master protocol trials, including platform trials, may be inflated due to variable registration methods [41,55], with some trials registering as one record [56,57] and others as multiple [58]. Nevertheless, we posit that ClinicalTrials.gov, the largest public clinical trial registry in the world [59], is the most appropriate data source for this analysis.

Most 2023 trials in our dataset were ongoing at the time of data download (05/01/2024), so enrollment figures largely reflect anticipated rather than actual numbers. Anticipated enrollment typically exceeds actual enrollment, meaning that our 2023 cohort estimates may overstate trial size. This would reinforce our conclusion about the proportion of small trials and minimal landscape change when compared to the 2018 or 2007–2010 analyses. However, this limitation should be considered when interpreting our findings.

Additionally, our study’s methodologies differ in some ways from those of the 2012 study [12]. The prior study evaluated all interventional trials registered in ClinicalTrials.gov [12], whereas our analysis was restricted to interventional trials with at least one US site. In addition, policies enacted since 2012, such as the NIH Policy on the Dissemination of NIH-Funded Clinical Trial Information, have increased the scope of trials required to be registered in ClinicalTrials.gov and improved reporting requirements [54].

### Conclusion

Despite increasing demands for reform, the current interventional clinical trial ecosystem continues to be dominated by small trials, and many trials lack a control group and are incomplete within five years. Policies that incentivize modern trial designs and a national research-ready infrastructure should be considered to improve the US evidence generation system’s ability to efficiently address pressing public health concerns.

## Supporting information

10.1017/cts.2026.10701.sm001 Sullenger et al. supplementary material Sullenger et al. supplementary material

## Supplementary material

The supplementary material for this article can be found at https://doi.org/10.1017/cts.2026.10701.

## Data access, responsibility, and analysis declaration

Karen E. Chiswell had full access to all the data in the study and takes responsibility for the integrity of the data and the accuracy of the data analysis.
