# Supplementary material for: Characteristics of interventional clinical trials registered in ClinicalTrials.gov, 2018–2023
Source: J Clin Transl Sci. 2026 Mar 25;10(1):e66. doi: 10.1017/cts.2026.10701 (PMC13107079; doi:10.1017/cts.2026.10701)
Supplement: Sullenger et al. supplementary material [file S2059866126107018sup001.docx]

**Supplement 1**

eFigure 1: Identification of sample among trials started in 2018

eFigure 2: Identification of sample among trials started in 2023

eTable 1: Derivation of Funding Source

eTable 2: Characteristics of Interventional Clinical Trials Started in 2023 and Registered in ClinicalTrials.gov by Therapeutic Area

eTable 3: Five-Year Follow-Up Characteristics of Phase 3 Interventional Clinical Trials Started in 2018 and Registered in ClinicalTrials.gov by Funding Source

**eFigure 1: Identification of sample among trials started in 2018**


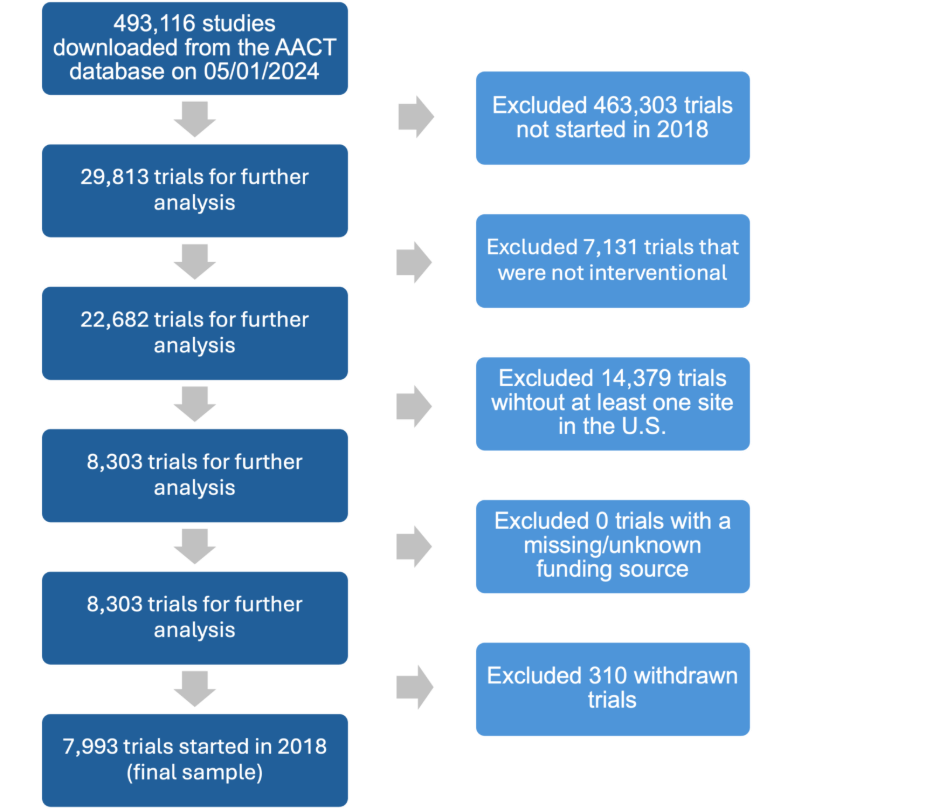


**eFigure 2: Identification of sample among trials started in 2023**


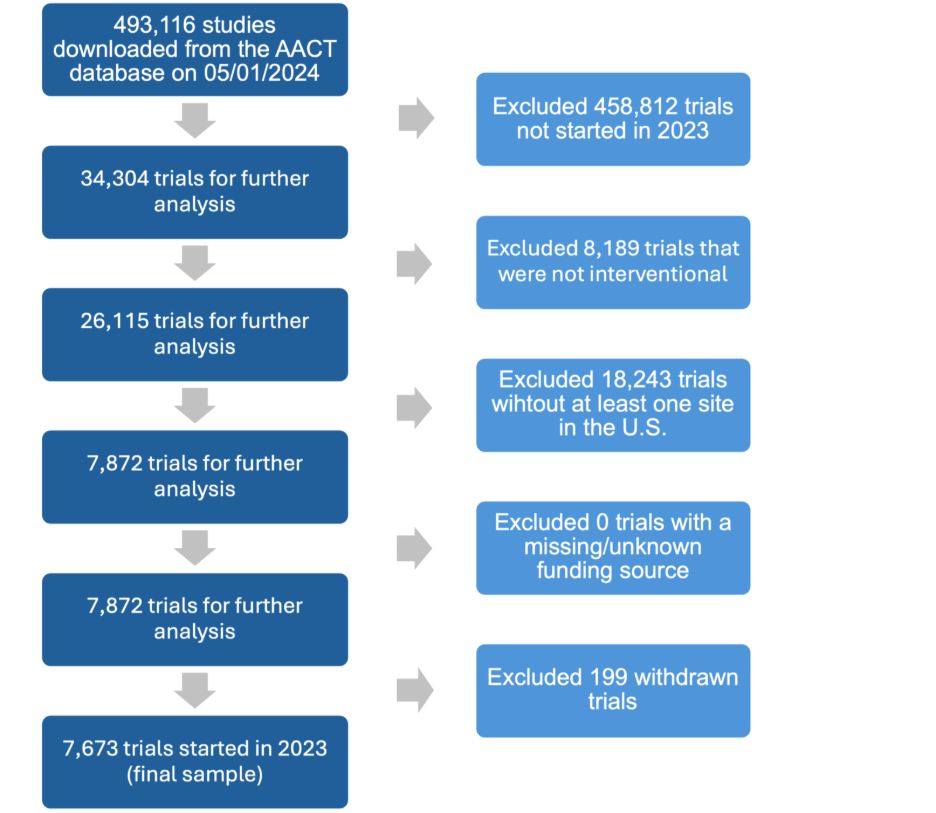


**eTable 1: Derivation of Funding Source**

| **Funding Source** | **Lead Sponsor** | **Collaborators** |
| --- | --- | --- |
| Industry | Industry | Any type |
|  | Other | At least one from Industry, none from NIH/Other U.S. Fed |
| NIH | NIH | Any type |
|  | Other U.S. Fed | At least one from NIH |
|  | Other | At least one from NIH |
| Other U.S. Fed | Other U.S. Fed | None from NIH |
|  | Other | At least one from Other U.S. Fed, none from NIH |
| Other | Other | None from Industry, NIH, Other U.S. Fed |

**eTable 2: Characteristics of Interventional Clinical Trials Started in 2023 and Registered in ClinicalTrials.gov by Therapeutic Area**

|  | | **Therapeutic Area** | | |
| --- | --- | --- | --- | --- |
|  | **All studies N=7,673** | **Cancer N=1,768** | **Cardiovascular N=790** | **Mental Health N=1,488** |
| Enrollment (categorical), n/N (%) |  |  |  |  |
| 1-100 | 4,872 (63.5%) | 1,163 (65.8%) | 484 (61.3%) | 911 (61.2%) |
| 101-500 | 2,113 (27.5%) | 467 (26.4%) | 203 (25.7%) | 449 (30.2%) |
| 501-1000 | 360 (4.7%) | 77 (4.4%) | 51 (6.5%) | 57 (3.8%) |
| >1000 | 328 (4.3%) | 61 (3.5%) | 52 (6.6%) | 71 (4.8%) |
| Enrollment (median) |  |  |  |  |
| Median (Q1, Q3) | 65 (30, 180) | 60 (30, 150) | 70 (30, 210) | 75 (40, 200) |
| Enrollment type, n/N (%) |  |  |  |  |
| Actual | 1,049 (13.7%) | 72 (4.1%) | 72 (9.1%) | 188 (12.6%) |
| Anticipated | 6,624 (86.3%) | 1,696 (95.9%) | 718 (90.9%) | 1,300 (87.4%) |
| Completion status, n/N (%) |  |  |  |  |
| Not yet recruiting | 313 (4.1%) | 42 (2.4%) | 41 (5.2%) | 68 (4.6%) |
| Recruiting | 5,646 (73.6%) | 1,552 (87.8%) | 600 (75.9%) | 1,090 (73.3%) |
| Enrolling by invitation | 376 (4.9%) | 34 (1.9%) | 47 (5.9%) | 90 (6.0%) |
| Active, not recruiting | 534 (7.0%) | 78 (4.4%) | 50 (6.3%) | 98 (6.6%) |
| Completed | 692 (9.0%) | 26 (1.5%) | 40 (5.1%) | 122 (8.2%) |
| Suspended | 49 (0.6%) | 21 (1.2%) | 7 (0.9%) | 8 (0.5%) |
| Terminated | 62 (0.8%) | 15 (0.8%) | 5 (0.6%) | 11 (0.7%) |
| Unknown | 1 (0.0%) | 0 (0.0%) | 0 (0.0%) | 1 (0.1%) |
| Intervention types^a^, n/N (%) |  |  |  |  |
| Drug | 3,009 (39.2%) | 1,078 (61.0%) | 239 (30.3%) | 288 (19.4%) |
| Device | 1,189 (15.5%) | 111 (6.3%) | 209 (26.5%) | 223 (15.0%) |
| Biological or vaccine | 480 (6.3%) | 238 (13.5%) | 24 (3.0%) | 13 (0.9%) |
| Procedure or surgery | 453 (5.9%) | 258 (14.6%) | 33 (4.2%) | 15 (1.0%) |
| Behavioral | 1,978 (25.8%) | 234 (13.2%) | 185 (23.4%) | 822 (55.2%) |
| Dietary supplement | 242 (3.2%) | 15 (0.8%) | 27 (3.4%) | 34 (2.3%) |
| Genetic | 34 (0.4%) | 7 (0.4%) | 5 (0.6%) | 0 (0.0%) |
| Radiation | 137 (1.8%) | 125 (7.1%) | 3 (0.4%) | 1 (0.1%) |
| Combination product | 80 (1.0%) | 21 (1.2%) | 8 (1.0%) | 9 (0.6%) |
| Diagnostic test | 124 (1.6%) | 46 (2.6%) | 21 (2.7%) | 4 (0.3%) |
| Other | 1,430 (18.6%) | 292 (16.5%) | 164 (20.8%) | 273 (18.3%) |
| Study phase (for studies with drug interventions^b^), n/N (%) |  |  |  |  |
| Early Phase 1/Phase 1 | 1,037/3,302 (31.4%) | 422/1,188 (35.5%) | 52/247 (21.1%) | 80/287 (27.9%) |
| Phase 1/2 | 355/3,302 (10.8%) | 208/1,188 (17.5%) | 22/247 (8.9%) | 14/287 (4.9%) |
| Phase 2 | 1,003/3,302 (30.4%) | 406/1,188 (34.2%) | 89/247 (36.0%) | 104/287 (36.2%) |
| Phase 2/3 | 82/3,302 (2.5%) | 20/1,188 (1.7%) | 8/247 (3.2%) | 13/287 (4.5%) |
| Phase 3 | 517/3,302 (15.7%) | 118/1,188 (9.9%) | 43/247 (17.4%) | 34/287 (11.8%) |
| Phase 4 | 308/3,302 (9.3%) | 14/1,188 (1.2%) | 33/247 (13.4%) | 42/287 (14.6%) |
| Interventional model, n/N (%) |  |  |  |  |
| Single Group | 2,210 (28.8%) | 737 (41.7%) | 236 (29.9%) | 345 (23.2%) |
| Parallel | 4,091 (53.3%) | 668 (37.8%) | 430 (54.4%) | 923 (62.0%) |
| Crossover | 597 (7.8%) | 12 (0.7%) | 69 (8.7%) | 138 (9.3%) |
| Sequential | 652 (8.5%) | 336 (19.0%) | 41 (5.2%) | 49 (3.3%) |
| Factorial | 123 (1.6%) | 15 (0.8%) | 14 (1.8%) | 33 (2.2%) |
| Multi-site study, n/N (%) | 2,625 (34.2%) | 849 (48.0%) | 280 (35.4%) | 284 (19.1%) |
| Locations of study sites, n/N (%) |  |  |  |  |
| U.S. only | 6,752 (88.0%) | 1,436 (81.2%) | 694 (87.8%) | 1,447 (97.2%) |
| U.S. and rest of the world | 921 (12.0%) | 332 (18.8%) | 96 (12.2%) | 41 (2.8%) |
| Randomized (for studies with >1 arms), n/N (%) | 4,728/5,565 (85.0%) | 650/1,025 (63.4%) | 508/571 (89.0%) | 1,105/1,170 (94.4%) |
| Number of parties masked (for randomized studies with >1 arm), n/N (%) |  |  |  |  |
| No masking | 1,725/4,728 (36.5%) | 449/650 (69.1%) | 184/508 (36.2%) | 375/1,105 (33.9%) |
| 1 party masked | 978/4,728 (20.7%) | 83/650 (12.8%) | 112/508 (22.0%) | 299/1,105 (27.1%) |
| 2 or more parties masked | 2,025/4,728 (42.8%) | 58/650 (18.1%) | 83/508 (41.7%) | 202/1,105 (39.0%) |
| DMC appointed (for randomized studies with >1 arm), n/N (%) |  |  |  |  |
| Yes | 1,867/4,728 (39.5%) | 358/650 (55.1%) | 229/508 (45.1%) | 443/1,105 (40.1%) |
| No | 2,291/4,728 (48.5%) | 237/650 (36.5%) | 228/508 (44.9%) | 543/1,105 (49.1%) |
| Unknown | 570/4,728 (12.1%) | 55/650 (8.5%) | 51/508 (10.0%) | 119/1,105 (10.8%) |
| Plan to share individual participant data, n/N (%) |  |  |  |  |
| Yes | 1,678/6,003 (28.0%) | 407/1,228 (33.1%) | 146/632 (23.1%) | 402/1,214 (33.1%) |
| No | 3,969/6,003 (66.1%) | 748/1,228 (60.9%) | 453/632 (71.7%) | 743/1,214 (61.2%) |
| Undecided | 356/6,003 (5.9%) | 73/1,228 (5.9%) | 33/632 (5.2%) | 69/1,214 (5.7%) |

1. Rows are not mutually exclusive. A study may be counted in more than one row.
2. Studies a U.S. FDA-regulated Drug Product. A “US FDA-regulated drug or biological product” is a separate field in ClinicalTrials.gov in which trial investigators are able to “Indicate whether this study includes an intervention subject to U.S. Food and Drug Administration regulation under section 351 of the Public Health Service Act or any of the following sections of the Federal Food, Drug, and Cosmetic Act: 505, 510(k), 515, 520(m), and 522. Select Yes/No.”

**eTable 3: Five-Year Follow-Up Characteristics of Phase 3 Interventional Clinical Trials Started in 2018 and Registered in ClinicalTrials.gov by Funding Source**

|  | | **Funder Type** | | | |
| --- | --- | --- | --- | --- | --- |
|  | **All studies N=677** | **Industry N=582** | **NIH N=26** | **Other U.S Fed N=8** | **Other N=61** |
| Enrollment, n/N (%) |  |  |  |  |  |
| 1-100 | 154 (22.7%) | 107 (18.4%) | 4 (15.4%) | 6 (75.0%) | 37/61 (60.7%) |
| 101-500 | 296 (43.7%) | 263 (45.2%) | 13 (50.0%) | 2 (25.0%) | 18/61 (29.5%) |
| 501-1000 | 151 (22.3%) | 143 (24.6%) | 5 (19.2%) | 0 (0.0%) | 3/61 (4.9%) |
| >1000 | 76 (11.2%) | 69 (11.9%) | 4 (15.4%) | 0 (0.0%) | 3/61 (4.9%) |
| Enrollment type, n/N (%) |  |  |  |  |  |
| Actual | 597 (88.2%) | 526 (90.4%) | 17 (65.4%) | 7 (87.5%) | 47 (77.0%) |
| Anticipated | 80 (11.8%) | 56 (9.6%) | 9 (34.6%) | 1 (12.5%) | 14 (23.0%) |
| Completion status, n/N (%) |  |  |  |  |  |
| Not yet recruiting | 0 (0.0%) | 0 (0.0%) | 0 (0.0%) | 0 (0.0%) | 0/61 (0.0%) |
| Recruiting | 39 (5.8%) | 26 (4.5%) | 5 (19.2%) | 1 (12.5%) | 7/61 (11.5%) |
| Enrolling by invitation | 4 (0.6%) | 2 (0.3%) | 0 (0.0%) | 0 (0.0%) | 2/61 (3.3%) |
| Active, not recruiting | 103 (15.2%) | 88 (15.1%) | 6 (23.1%) | 2 (25.0%) | 7/61 (11.5%) |
| Completed | 402 (59.4%) | 356 (61.2%) | 11 (42.3%) | 2 (25.0%) | 33/61 (54.1%) |
| Suspended | 4 (0.6%) | 1 (0.2%) | 1 (3.8%) | 0 (0.0%) | 2/61 (3.3%) |
| Terminated | 113 (16.7%) | 100 (17.2%) | 3 (11.5%) | 3 (37.5%) | 7/61 (11.5%) |
| Unknown | 12 (1.8%) | 9 (1.5%) | 0 (0.0%) | 0 (0.0%) | 3/61 (4.9%) |
| Therapeutic area^a,b^, n/N (%) |  |  |  |  |  |
| Cancer | 142 (21.0%) | 119 (20.4%) | 13 (50.0%) | 1 (12.5%) | 9/61 (14.8%) |
| Cardiovascular | 62 (9.2%) | 50 (8.6%) | 3 (11.5%) | 1 (12.5%) | 8/61 (13.1%) |
| Mental Health | 53 (7.8%) | 42 (7.2%) | 3 (11.5%) | 2 (25.0%) | 6/61 (9.8%) |
| Intervention types^a^, n/N (%) |  |  |  |  |  |
| Drug | 598 (88.3%) | 511 (87.8%) | 21 (80.8%) | 8 (100.0%) | 58/61 (95.1%) |
| Device | 11 (1.6%) | 9 (1.5%) | 0 (0.0%) | 0 (0.0%) | 2/61 (3.3%) |
| Biological or vaccine | 88 (13.0%) | 79 (13.6%) | 8 (30.8%) | 0 (0.0%) | 1/61 (1.6%) |
| Procedure or surgery | 22 (3.2%) | 11 (1.9%) | 8 (30.8%) | 0 (0.0%) | 3/61 (4.9%) |
| Behavioral | 8 (1.2%) | 1 (0.2%) | 2 (7.7%) | 1 (12.5%) | 4/61 (6.6%) |
| Dietary supplement | 1 (0.1%) | 1 (0.2%) | 0 (0.0%) | 0 (0.0%) | 0/61 (0.0%) |
| Genetic | 5 (0.7%) | 4 (0.7%) | 0 (0.0%) | 0 (0.0%) | 1/61 (1.6%) |
| Radiation | 10 (1.5%) | 5 (0.9%) | 3 (11.5%) | 0 (0.0%) | 2/61 (3.3%) |
| Combination product | 14 (2.1%) | 13 (2.2%) | 0 (0.0%) | 0 (0.0%) | 1/61 (1.6%) |
| Diagnostic test | 4 (0.6%) | 3 (0.5%) | 1 (3.8%) | 0 (0.0%) | 0/61 (0.0%) |
| Other | 90 (13.3%) | 68 (11.7%) | 13 (50.0%) | 0 (0.0%) | 9/61 (14.8%) |
| Interventional model, n/N (%) |  |  |  |  |  |
| Single Group | 98 (14.5%) | 91 (15.6%) | 0 (0.0%) | 0 (0.0%) | 7/61 (11.5%) |
| Parallel | 554 (81.8%) | 470 (80.8%) | 26 (100.0%) | 7 (87.5%) | 51/61 (83.6%) |
| Crossover | 19 (2.8%) | 16 (2.7%) | 0 (0.0%) | 1 (12.5%) | 2/61 (3.3%) |
| Sequential | 5 (0.7%) | 5 (0.9%) | 0 (0.0%) | 0 (0.0%) | 0/61 (0.0%) |
| Factorial | 1 (0.1%) | 0 (0.0%) | 0 (0.0%) | 0 (0.0%) | 1/61 (1.6%) |
| Multi-site study, n/N (%) | 584 (86.3%) | 538 (92.4%) | 20 (76.9%) | 2 (25.0%) | 24/61 (39.3%) |
| Locations of study sites, n/N (%) |  |  |  |  |  |
| U.S. only | 264 (39.0%) | 182 (31.3%) | 18 (69.2%) | 8 (100.0%) | 56/61 (91.8%) |
| U.S. and rest of the world | 413 (61.0%) | 400 (68.7%) | 8 (30.8%) | 0 (0.0%) | 5/61 (8.2%) |
| Randomized (for studies with >1 arms), n/N (%) | 563/586 (96.1%) | 479/498 (96.2%) | 25 (96.2%) | 8 (100.0%) | 51/54 (94.4%) |
| Number of parties masked (for randomized studies with >1 arm), n/N (%) |  |  |  |  |  |
| No masking | 106/563 (18.8%) | 86/479 (18.0%) | 9/25 (36.0%) | 0 (0.0%) | 11/51 (21.6%) |
| 1 party masked | 25/563 (4.4%) | 17/479 (3.5%) | 1/25 (4.0%) | 2 (25.0%) | 5/51 (9.8%) |
| 2 or more parties masked | 432/563 (76.7%) | 376/479 (78.5%) | 15/25 (60.0%) | 6 (75.0%) | 35/51 (68.6%) |
| DMC appointed (for randomized studies with >1 arm), n/N (%) |  |  |  |  |  |
| Yes | 323/563 (57.4%) | 271/479 (56.6%) | 18/25 (72.0%) | 4 (50.0%) | 30/51 (58.8%) |
| No | 191/563 (33.9%) | 165/479 (34.4%) | 7/25 (28.0%) | 2 (25.0%) | 17/51 (33.3%) |
| Unknown | 49/563 (8.7%) | 43/479 (9.0%) | 0/25 (0.0%) | 2 (25.0%) | 4/51 (7.8%) |

1. Rows are not mutually exclusive. A study may be counted in more than one row.
2. Conditions grouped as in 2012 Califf paper, supplemented with clinical review of new terms occurring in 5 or more trials. Condition groupings are not exhaustive.

**Supplement 2: Therapeutic Area Terms**

| **Cardiovascular** | **Mental Health** | **Cancer** |
| --- | --- | --- |
| acute aortic syndrome | aberrant motor behavior in dementia | adenocarcinoma of the pancreas |
| aortic aneurysm, thoracoabdominal | addiction | abdominal cancer |
| aortic dissection | adhd - combined type | accelerated phase chronic myelogenous leukemia, bcr-abl1 positive |
| atherosclerotic cardiovascular disease | adolescent - emotional problem | acute biphenotypic leukemia |
| cardiac amyloidosis | adolescent problem behavior | acute lymphoblastic leukemia in remission |
| cardiac arrhythmia | agitation in patients with dementia of the alzheimer's type | acute lymphoid leukemia |
| cardiac event | alcohol use disorder | acute myeloid leukemia arising from previous myelodysplastic syndrome |
| cardiac failure | alcohol use disorder (aud) | acute myeloid leukemia in remission |
| cardiac sarcoidosis | alcohol; use, problem | acute myeloid leukemia post cytotoxic therapy |
| cardiac transplant disorder | alzheimer dementia | acute myeloid leukemia refractory |
| cardiogenic shock | alzheimer disease, early onset | acute myeloid leukemia, in relapse |
| cardiometabolic syndrome | alzheimer disease, late onset | acute undifferentiated leukemia |
| cardiovascular health | alzheimer's disease (incl subtypes) | adenoid cystic carcinoma |
| cardiovascular risk factor | alzheimer's disease and related dementias | adult solid tumor |
| cerebral vascular accident | alzheimers disease | advanced breast carcinoma |
| chronic stroke | anxiety and fear | advanced cholangiocarcinoma |
| chronic thromboembolic pulmonary hypertension | anxiety depression | advanced clear cell renal cell carcinoma |
| congenital heart defect | anxiety disorders and symptoms | advanced colorectal carcinoma |
| congenital heart disease in children | anxiety generalized | advanced endometrial carcinoma |
| cva | anxiety sensitivity | advanced gastroesophageal junction adenocarcinoma |
| cva (cerebrovascular accident) | anxiety state | advanced head and neck squamous cell carcinoma |
| dissection, blood vessel | arachnophobia | advanced lung carcinoma |
| functional mitral regurgitation | asd | advanced lung non-small cell carcinoma |
| heart attack | atypical anorexia nervosa | advanced lymphoma |
| heart failure nyha class ii | autism spectrum disorder (asd) | advanced malignant neoplasm |
| heart failure nyha class iii | avoidant restrictive food intake disorder | advanced malignant solid neoplasm |
| heart failure nyha class iv | avoidant/restrictive food intake disorder | advanced ovarian carcinoma |
| heart failure with normal ejection fraction | behavior disorders | advanced pancreatic adenocarcinoma |
| heart failure with preserved ejection fraction | behavior problem | advanced pancreatic cancer |
| heart failure with preserved ejection fraction (hfpef) | binge eating | advanced pancreatic carcinoma |
| heart failure with reduced ejection fraction | bipolar disorder i | advanced prostate cancer |
| heart failure with reduced ejection fraction (hfref) | bipolar ii disorder | advanced prostate carcinoma |
| heart failure; with decompensation | body image disturbance | advanced solid tumors cancer |
| heart failureÔºåcongestive | cannabis use disorder | advanced urothelial carcinoma |
| heart rate variability | caregiver stress | advanced/metastatic solid tumors |
| heart transplant | caregiver stress syndrome | aggressive non-hodgkin lymphoma |
| hemorrhagic stroke | cbt | aml, adult |
| hypotension and shock | child behavior problem | ampullary cancer |
| icd | cocaine use disorder | anaplastic ependymoma |
| implantable defibrillator user | delirium in old age | anatomic stage 0 breast cancer ajcc v8 |
| mitral valve disease | dementia alzheimers | anatomic stage i breast cancer ajcc v8 |
| myocardial fibrosis | dementia moderate | anatomic stage ia breast cancer ajcc v8 |
| nstemi - non-st segment elevation mi | dementia of alzheimer type | anatomic stage ib breast cancer ajcc v8 |
| pah | dementia, alzheimer type | anatomic stage ii breast cancer ajcc v8 |
| paroxysmal supraventricular tachycardia | dementia, mild | anatomic stage iia breast cancer ajcc v8 |
| patent foramen ovale | dementia, mixed | anatomic stage iib breast cancer ajcc v8 |
| raynaud phenomenon | depression in adolescence | anatomic stage iii breast cancer ajcc v8 |
| right heart failure | depression in old age | anatomic stage iiia breast cancer ajcc v8 |
| right ventricular dysfunction | depression mild | anatomic stage iiib breast cancer ajcc v8 |
| single-ventricle | depression, anxiety | anatomic stage iiic breast cancer ajcc v8 |
| stemi | depression, unipolar | anatomic stage iv breast cancer ajcc v8 |
| stroke hemorrhagic | depressive episode | angiosarcoma |
| stroke sequelae | distress, emotional | appendix cancer |
| stroke, ischemic | drug use disorders | arterial occlusion |
| systolic heart failure | early alzheimer's disease | arterial stiffness |
| thoracoabdominal aortic aneurysm | eating disorder | astrocytoma, grade iii |
| tia | eating disorder symptom | atypical teratoid/rhabdoid tumor |
| tricuspid regurgitation | eating disorders in adolescence | b acute lymphoblastic leukemia |
| tricuspid valve disease | emotion regulation | b cell lymphoma |
| univentricular heart | emotional distress | b-all |
| vascular dilation | emotional disturbances | b-cell acute lymphoblastic leukemia |
| aortic rupture | emotional dysfunction | b-cell non hodgkin lymphoma |
| aortitis | emotional regulation | b-cell non-hodgkin lymphoma |
| carcinoid heart disease | emotional stress | bclc stage b hepatocellular carcinoma |
| stroke | excoriation disorder | bclc stage c hepatocellular carcinoma |
| abdominal aortic aneurysm | gulf war illness | biochemically recurrent prostate carcinoma |
| abdominal aortic aneurysms | gulf war syndrome | bladder carcinoma |
| ablation techniques | insomnia disorder | bladder urothelial carcinoma |
| accelerated idioventricular rhythm | mdd | blast phase chronic myelogenous leukemia, bcr-abl1 positive |
| acute coronary syndrome | medication assisted treatment | blastic plasmacytoid dendritic cell neoplasm |
| acute coronary syndromes | mental disorder | blood cancer |
| acute decompensated heart failure | mental disorder in adolescence | bone cancer |
| acute heart failure | mental disorders, severe | bone sarcoma |
| acute ischemic stroke | mental health disorder | borderline resectable pancreatic adenocarcinoma |
| acute myocardial infarction | mental health impairment | brain metastases, adult |
| acute stroke | mental health issue | brain tumor, pediatric |
| adams-stokes syndrome | mental health wellness | brain tumor, recurrent |
| advanced cardiac life support | mental health wellness 1 | breast adenocarcinoma |
| advanced heart failure | mental illness | breast cancer female |
| andersen syndrome | mental stress | breast cancer metastatic |
| aneurysm | methamphetamine abuse | breast cancer screening |
| aneurysm, dissecting | methamphetamine use disorder | breast cancer stage i |
| aneurysm, false | methamphetamine-dependence | breast cancer stage ii |
| aneurysm, ruptured | mild dementia | breast cancer stage iii |
| angina | misophonia | breast cancer stage iv |
| angina pectoris | mood disturbance | breast cancer survivors |
| angina pectoris, variant | narcotic-related disorders | breast ductal carcinoma in situ |
| angina, unstable | nicotine addiction | breast inflammatory carcinoma |
| angiocardiography | nicotine dependence, cigarettes | breast lobular carcinoma in situ |
| angiography | nicotine use disorder | breast neoplasm female |
| angioplasty | ocd | breast tumor |
| angioplasty, balloon | opioid addiction | cancer colorectal |
| angioplasty, balloon, laser-assisted | opioid use disorder | cancer metastatic |
| angioplasty, laser | opioid use disorder, severe | cancer of breast |
| angioplasty, transluminal, percutaneous coronary | opioid-use disorder | cancer of head and neck |
| ankle brachial index | parkinson disease dementia | cancer of pancreas |
| anterior wall myocardial infarction | perinatal depression | cancer of prostate |
| aortic aneurysm | physiological stress | cancer of the pancreas |
| aortic aneurysm, abdominal | post intensive care unit syndrome | cancer-related problem/condition |
| aortic aneurysm, thoracic | post partum depression | cancer, breast |
| aortic arch syndromes | postpartum anxiety | cancer, lung |
| aortic coarctation | postpartum disorder | carcinoma, squamous cell of head and neck |
| aortic diseases | prenatal stress | castrate resistant prostate cancer |
| aortic stenosis | procedural anxiety | castration resistant prostatic cancer |
| aortic stenosis, subvalvular | prodromal alzheimer's disease | castration-resistant prostate cancer |
| aortic stenosis, supravalvular | prodromal schizophrenia | castration-resistant prostate carcinoma |
| aortic valve disease | prolonged grief disorder | castration-sensitive prostate carcinoma |
| aortic valve insufficiency | psychiatric disorder | central nervous system lymphoma |
| aortic valve prolapse | psychological distress | central nervous system tumor |
| aortic valve stenosis | psychological stress | cervical carcinoma |
| aortography | psychosis nos/other | cervix cancer |
| aortopulmonary septal defect | rumination syndrome | childhood cancer |
| arrhythmia | schizo affective disorder | childhood cancer survivors |
| arrhythmia, sinus | schizophrenia and related disorders | choroid plexus carcinoma |
| arrhythmias, cardiac | schizophrenia, treatment-resistant | chromophobe renal cell carcinoma |
| arrhythmogenic right ventricular dysplasia | schizophrenia; psychosis | chronic lymphocytic leukemia/small lymphocytic lymphoma |
| arterial hypertension | schizophreniform disorders | chronic myelogenous leukemia, bcr-abl1 positive |
| arterial occlusive disease | smoking (tobacco) addiction | chronic phase chronic myelogenous leukemia, bcr-abl1 positive |
| arterial occlusive diseases | social anxiety | cigarette smoking-related carcinoma |
| arteriolosclerosis | social anxiety disorder of childhood | classical hodgkin lymphoma |
| arteriosclerosis | social stress | clear cell sarcoma |
| arteriosclerosis obliterans | specific phobia | clinical stage i cutaneous melanoma ajcc v8 |
| assisted circulation | stimulant use disorder | clinical stage i gastric cancer ajcc v8 |
| atherectomy | stress disorder | clinical stage i gastroesophageal junction adenocarcinoma ajcc v8 |
| atherectomy, coronary | stress reaction | clinical stage i hpv-mediated (p16-positive) oropharyngeal carcinoma ajcc v8 |
| atherosclerosis | stress, emotional | clinical stage ii cutaneous melanoma ajcc v8 |
| atrial fibrillation | substance use disorder | clinical stage ii esophageal adenocarcinoma ajcc v8 |
| atrial flutter | trauma, psychological | clinical stage ii gastroesophageal junction adenocarcinoma ajcc v8 |
| atrial function | trichotillomania (hair-pulling disorder) | clinical stage ii hpv-mediated (p16-positive) oropharyngeal carcinoma ajcc v8 |
| atrial function, left | vascular dementia | clinical stage iia esophageal adenocarcinoma ajcc v8 |
| atrial function, right | work related stress | clinical stage iia gastroesophageal junction adenocarcinoma ajcc v8 |
| atrial premature complexes | aids dementia complex | clinical stage iib esophageal adenocarcinoma ajcc v8 |
| atrioventricular block | alzheimer disease | clinical stage iib gastroesophageal junction adenocarcinoma ajcc v8 |
| auscultation | dementia | clinical stage iii cutaneous melanoma ajcc v8 |
| ballistocardiography | dementia, multi-infarct | clinical stage iii esophageal adenocarcinoma ajcc v8 |
| balloon dilatation | dementia, vascular | clinical stage iii gastric cancer ajcc v8 |
| blood circulation | emotions | clinical stage iii gastroesophageal junction adenocarcinoma ajcc v8 |
| blood circulation time | frontotemporal dementia | clinical stage iii hpv-mediated (p16-positive) oropharyngeal carcinoma ajcc v8 |
| blood pressure | heroin dependence | clinical stage iv cutaneous melanoma ajcc v8 |
| bradycardia | morphine dependence | clinical stage iv esophageal adenocarcinoma ajcc v8 |
| bundle-branch block | abreaction | clinical stage iv gastric cancer ajcc v8 |
| capillary resistance | adhd | clinical stage iv gastroesophageal junction adenocarcinoma ajcc v8 |
| cardiac arrest | adjustment disorders | clinical stage iv hpv-mediated (p16-positive) oropharyngeal carcinoma ajcc v8 |
| cardiac catheterization | adolescent psychiatry | clinical stage iva esophageal adenocarcinoma ajcc v8 |
| cardiac complexes, premature | affect | clinical stage iva gastric cancer ajcc v8 |
| cardiac disease | affective disorders, psychotic | clinical stage iva gastroesophageal junction adenocarcinoma ajcc v8 |
| cardiac output | aggression | clinical stage ivb esophageal adenocarcinoma ajcc v8 |
| cardiac output, high | agitation | clinical stage ivb gastric cancer ajcc v8 |
| cardiac output, low | agoraphobia | clinical stage ivb gastroesophageal junction adenocarcinoma ajcc v8 |
| cardiac pacing, artificial | alcohol abuse | cll/sll |
| cardiac surgery | alcohol consumption | cmml |
| cardiac surgical procedures | alcohol dependence | cns tumor |
| cardiac tamponade | alcohol drinking | colo-rectal cancer |
| cardiac toxicity | altruism | colon adenocarcinoma |
| cardiac transplantation | alzheimer's disease | colon adenoma |
| cardiac volume | alzheimerazs disease | colon carcinoma |
| cardiac-gated imaging techniques | amnestic mild cognitive impairment | colorectal adenocarcinoma |
| cardiac-gated single-photon emission computer-assisted tomography | anger | colorectal adenoma |
| cardiomegaly | anorexia nervosa | colorectal cancer (crc) |
| cardiomyopathies | antisocial personality disorder | colorectal cancer metastatic |
| cardiomyopathy | anxiety | colorectal tumors |
| cardiomyopathy, dilated | anxiety disorder | crc |
| cardiomyopathy, hypertrophic | anxiety disorders | ctcl |
| cardiomyopathy, restrictive | anxiety, separation | cutaneous melanoma |
| cardiomyoplasty | apathy | cutaneous squamous cell carcinoma |
| cardiopulmonary bypass | asperger syndrome | cutaneous t cell lymphoma |
| cardiopulmonary resuscitation | asperger's disorder | dcis |
| cardiovascular abnormalities | association | diffuse glioma |
| cardiovascular deconditioning | association learning | diffuse intrinsic pontine glioma |
| cardiovascular disease | attention | diffuse large b cell lymphoma |
| cardiovascular diseases | attention deficit and disruptive behavior disorders | diffuse large b cell lymphoma (dlbcl) |
| cardiovascular infections | attention deficit disorder | diffuse large b-cell lymphoma (dlbcl) |
| cardiovascular physiological phenomena | attention deficit disorder with hyperactivity | diffuse large b-cell lymphoma, not otherwise specified |
| cardiovascular physiological processes | attention deficit hyperactivity disorder | diffuse midline glioma, h3 k27m-mutant |
| cardiovascular risk | attention deficit/hyperactivity disorder | digestive system neoplasm |
| cardiovascular risk factors | attention-deficit/hyperactivity disorder | dlbcl |
| cardiovascular surgical procedures | attentional blink | early breast cancer |
| carotid artery disease | autism | early-stage breast cancer |
| carotid artery stenosis | autism spectrum disorder | ebv-positive diffuse large b-cell lymphoma, not otherwise specified |
| catheter ablation | autism spectrum disorders | endometrial adenocarcinoma |
| catheterization, central venous | autistic disorder | endometrial carcinoma |
| catheterization, peripheral | aversive therapy | endometrial clear cell adenocarcinoma |
| catheterization, swan-ganz | avoidance learning | endometrial endometrioid adenocarcinoma |
| central venous pressure | behavior problems | endometrial serous adenocarcinoma |
| cerebral stroke | behavior therapy | endometrioid adenocarcinoma |
| cerebral vasospasm | behavior, addictive | er positive breast cancer |
| cerebrovascular accident | behavioral disciplines and activities | esophageal adenocarcinoma |
| cerebrovascular circulation | behavioral medicine | esophagogastric cancer |
| cerebrovascular stroke | behavioral research | esophagus cancer |
| chagas cardiomyopathy | behavioral sciences | estrogen receptor negative |
| chest pain | bereavement | estrogen receptor positive |
| chronic heart failure | binge eating disorder | estrogen receptor-positive breast cancer |
| circulatory arrest, deep hypothermia induced | binge-eating disorder | ewing sarcoma |
| collateral circulation | biological psychiatry | extensive stage lung small cell carcinoma |
| commotio cordis | bipolar | extensive stage small cell lung cancer |
| congenital heart disease | bipolar depression | extensive-stage small cell lung cancer |
| congestive heart failure | bipolar disorder | fallopian tube adenocarcinoma |
| cor triatriatum | bipolar i disorder | fallopian tube carcinosarcoma |
| coronary aneurysm | body dysmorphic disorder | fallopian tube endometrioid adenocarcinoma |
| coronary angiography | body dysmorphic disorders | fallopian tube high grade serous adenocarcinoma |
| coronary arteriosclerosis | body image | financial stress |
| coronary artery bypass | bonding, human-pet | follicular lymphoma (fl) |
| coronary artery bypass graft surgery | borderline personality disorder | gall bladder cancer |
| coronary artery bypass grafting | brief psychiatric rating scale | gallbladder carcinoma |
| coronary artery bypass surgery | bulimia nervosa | gastric adenocarcinoma |
| coronary artery bypass, off-pump | cannabis dependence | gastric carcinoma |
| coronary artery disease | capgras syndrome | gastroesophageal adenocarcinoma |
| coronary artery disease (cad) | catharsis | gastroesophageal cancer |
| coronary artery diseases | child behavior | gastroesophageal junction adenocarcinoma |
| coronary artery stenosis | child behavior disorders | gastroesophageal junction cancer |
| coronary atherosclerosis | child development disorders, pervasive | gastroesophageal-junction cancer |
| coronary circulation | child psychiatry | genitourinary cancer |
| coronary disease | choice behavior | germ cell tumor |
| coronary heart disease | chronic fatigue syndrome | gist |
| coronary occlusion | chronic insomnia | glioblastoma multiforme of brain |
| coronary restenosis | chronic pain | glioblastoma multiforme, adult |
| coronary stenosis | cigarette smoking | glioblastoma, idh-wildtype |
| coronary thrombosis | cocaine addiction | glioma of brain |
| coronary vasospasm | cocaine dependence | glioma, malignant |
| coronary vessel anomalies | cognition | gliomas |
| counterpulsation | cognition disorders | grade 1 follicular lymphoma |
| crisscross heart | cognitive decline | grade 2 follicular lymphoma |
| critical limb ischemia | cognitive dysfunction | grade 3a follicular lymphoma |
| death, sudden | cognitive impairment | grade 3b follicular lymphoma |
| death, sudden, cardiac | cognitive science | gynecologic cancer |
| deep vein thrombosis | cognitive therapy | hairy cell leukemia |
| deep venous thrombosis | cognitive/functional effects | hcc |
| defibrillators | combat disorders | head and neck carcinoma |
| dextrocardia | commitment of mentally ill | head and neck carcinoma of unknown primary |
| diagnostic techniques, cardiovascular | community mental health services | head and neck squamous cell carcinoma (hnscc) |
| diastole | community psychiatry | hematologic cancer |
| diastolic dysfunction | complex regional pain syndrome | hematologic malignancy |
| diastolic heart failure | compulsive behavior | hematopoietic and lymphatic system neoplasm |
| dilated cardiomyopathy | compulsive personality disorder | hematopoietic and lymphoid cell neoplasm |
| discrete subaortic stenosis | concept formation | hematopoietic and lymphoid system neoplasm |
| double outlet right ventricle | conditioning (psychology) | her2 negative breast carcinoma |
| drug-eluting stents | conditioning, classical | her2 positive breast carcinoma |
| ductus arteriosus, patent | conditioning, eyelid | her2-negative breast cancer |
| dyslipidaemia | conditioning, operant | her2-negative breast carcinoma |
| dyslipidemia | conduct disorder | her2-positive breast cancer |
| ebstein anomaly | conversion disorder | her2-positive breast carcinoma |
| echocardiography | convulsive therapy | her2-positive gastric cancer |
| echocardiography, doppler | counseling | her2-positive metastatic breast cancer |
| echocardiography, doppler, color | countertransference (psychology) | her2/neu negative |
| echocardiography, four-dimensional | couples therapy | high grade b-cell lymphoma |
| echocardiography, stress | criminal psychology | high grade b-cell lymphoma with myc and bcl2 and/or bcl6 rearrangements |
| echocardiography, three-dimensional | crisis intervention | high grade b-cell lymphoma with myc and bcl2 or bcl6 rearrangements |
| echocardiography, transesophageal | critical period (psychology) | high grade b-cell lymphoma, not otherwise specified |
| ectopia cordis | cues | high grade glioma |
| edema, cardiac | cyclothymic disorder | high grade ovarian serous adenocarcinoma |
| eisenmenger complex | decision making | high risk myelodysplastic syndrome |
| electrocardiogram pattern | deja vu | high-grade b-cell lymphoma |
| electrocardiography | delirium, dementia, amnestic, cognitive disorders | hnscc |
| electrocardiography, ambulatory | delusions | hormone receptor positive breast carcinoma |
| electrodes | dementia with lewy bodies | hormone receptor positive tumor |
| electrodes, implanted | dental anxiety | hormone receptor-positive breast cancer |
| electrophysiologic techniques, cardiac | dependent personality disorder | hormone receptor-positive breast carcinoma |
| endocardial cushion defects | depersonalization | hpv positive oropharyngeal squamous cell carcinoma |
| endocardial fibroelastosis | depression | hpv-related carcinoma |
| endocarditis | depressive disorder | hpv-related malignancy |
| endocarditis, bacterial | depressive disorder, major | hpv-related squamous cell carcinoma |
| endocarditis, subacute bacterial | depressive symptoms | human papillomavirus-related carcinoma |
| endomyocardial fibrosis | desensitization, psychologic | indolent b-cell non-hodgkin lymphoma |
| endothelial dysfunction | directive counseling | indolent lymphoma |
| endothelial function | discrimination (psychology) | indolent non-hodgkin lymphoma |
| epicardial mapping | discrimination learning | intrahepatic cholangiocarcinoma |
| essential hypertension | dissociative disorders | invasive breast carcinoma |
| extracorporeal circulation | distress | job stress |
| familial hypercholesterolemia | dreams | kaposi sarcoma |
| foramen ovale, patent | drinking behavior | kras mutation-related tumors |
| fractional flow reserve, myocardial | drug abuse | large b-cell lymphoma |
| gated blood-pool imaging | drug addiction | larynx cancer |
| heart aneurysm | drug dependence | leptomeningeal metastases |
| heart arrest | dysthymic disorder | leptomeningeal metastasis |
| heart arrest, induced | eating disorders | limited stage lung small cell carcinoma |
| heart auscultation | electroconvulsive therapy | liver and intrahepatic bile duct carcinoma |
| heart block | emergency services, psychiatric | localized prostate carcinoma |
| heart bypass, left | emotional intelligence | locally advanced bladder urothelial carcinoma |
| heart bypass, right | euphoria | locally advanced breast cancer |
| heart catheterization | excessive daytime sleepiness | locally advanced gastroesophageal junction adenocarcinoma |
| heart defects, congenital | executive function | locally advanced head and neck squamous cell carcinoma |
| heart disease | exhibitionism | locally advanced hepatocellular carcinoma |
| heart diseases | expressed emotion | locally advanced lung non-small cell carcinoma |
| heart failure | extraversion (psychology) | locally advanced malignant solid neoplasm |
| heart failure, congestive | eye movement desensitization reprocessing | locally advanced melanoma |
| heart failure, diastolic | factitious disorders | locally advanced or metastatic solid tumors |
| heart failure, systolic | family conflict | locally advanced oropharyngeal squamous cell carcinoma |
| heart function tests | family therapy | locally advanced pancreatic adenocarcinoma |
| heart massage | father-child relations | locally advanced pancreatic ductal adenocarcinoma |
| heart murmurs | fathers | locally advanced rectal carcinoma |
| heart rate | fear | locally advanced renal pelvis urothelial carcinoma |
| heart rupture | fetishism (psychiatric) | locally advanced solid tumor |
| heart rupture, post-infarction | firesetting behavior | locally advanced ureter urothelial carcinoma |
| heart septal defects | first episode psychosis | locally advanced urethral urothelial carcinoma |
| heart septal defects, atrial | forensic psychiatry | locally advanced urothelial carcinoma |
| heart septal defects, ventricular | free association | low grade glioma |
| heart sounds | freudian theory | low-grade glioma |
| heart transplantation | gambling | lung adenocarcinoma |
| heart valve disease | gender identity | lung cancer metastatic |
| heart valve diseases | generalization (psychology) | lung cancer stage iii |
| heart valve prolapse | generalization, response | lung cancer, non-small cell |
| heart valve prosthesis | generalization, stimulus | lung cancer, nonsmall cell |
| heart valve prosthesis implantation | generalized anxiety disorder | lung carcinoma |
| heart-assist devices | genetics, behavioral | lung neoplasm |
| heart-lung machine | geriatric psychiatry | lung neoplasm malignant |
| heart, artificial | gestalt theory | lung non-small cell carcinoma |
| hemodynamics | gestalt therapy | lymphoplasmacytic lymphoma |
| high blood pressure | grief | malignant brain neoplasm |
| hirudin therapy | habits | malignant digestive system neoplasm |
| hypertension, pulmonary | happiness | malignant female reproductive system neoplasm |
| hypertrophic cardiomyopathy | harm reduction | malignant genitourinary system neoplasm |
| hypertrophy | helplessness, learned | malignant neoplasm |
| hypertrophy, left ventricular | histrionic personality disorder | malignant neoplasm of breast |
| hypertrophy, right ventricular | hostility | malignant neoplastic disease |
| hypoplastic left heart syndrome | human engineering | malignant peripheral nerve sheath tumors |
| hypotension | hypoactive sexual desire disorder | malignant solid neoplasm |
| iliac aneurysm | hypochondriasis | malignant solid tumor |
| in-stent restenosis | hypoxic-ischemic encephalopathy | mantle cell lymphoma (mcl) |
| infarction | hysteria | marginal zone lymphoma (mzl) |
| infective endocarditis | implosive therapy | mastectomy; lymphedema |
| inferior wall myocardial infarction | impotence | mcrpc |
| injections, intraventricular | imprinting (psychology) | melanoma stage iii |
| internal mammary-coronary artery anastomosis | impulse control disorders | melanoma stage iv |
| intra-aortic balloon pumping | impulsive behavior | melanoma, cutaneous malignant |
| ischemia | infant behavior | merkel cell carcinoma |
| ischemia reperfusion injury | inhibition (psychology) | mesothelioma, malignant |
| ischemic cardiomyopathy | insomnia | metastatic |
| ischemic heart disease | interpersonal relations | metastatic adenoid cystic carcinoma |
| ischemic preconditioning | irritable mood | metastatic bladder urothelial carcinoma |
| ischemic preconditioning, myocardial | jealousy | metastatic breast carcinoma |
| ischemic stroke | judgment | metastatic castration resistant prostate cancer |
| isolated noncompaction of the ventricular myocardium | jungian theory | metastatic castration-resistant prostate cancer |
| jervell-lange nielsen syndrome | koro | metastatic castration-resistant prostate cancer (mcrpc) |
| kinetocardiography | love | metastatic cervical cancer |
| left ventricular dysfunction | major depression | metastatic cholangiocarcinoma |
| left ventricular hypertrophy | major depressive disorder | metastatic clear cell renal cell carcinoma |
| levocardia | major depressive disorder (mdd) | metastatic colon adenocarcinoma |
| long qt syndrome | major depressive episode | metastatic colon cancer |
| lown-ganong-levine syndrome | malingering | metastatic colon carcinoma |
| lutembacher syndrome | man-machine systems | metastatic colorectal adenocarcinoma |
| magnetic resonance angiography | mania | metastatic colorectal carcinoma |
| magnetocardiography | marijuana dependence | metastatic endometrial carcinoma |
| microcirculation | marijuana smoking | metastatic esophageal adenocarcinoma |
| microscopic angioscopy | marital therapy | metastatic gastric adenocarcinoma |
| microvascular angina | masochism | metastatic gastric carcinoma |
| mitral regurgitation | masturbation | metastatic gastroesophageal junction adenocarcinoma |
| mitral valve insufficiency | maternal behavior | metastatic head and neck cancer |
| mitral valve prolapse | maternal-fetal relations | metastatic head and neck squamous cell carcinoma |
| mitral valve regurgitation | maze learning | metastatic head-and-neck squamous-cell carcinoma |
| mitral valve stenosis | medication adherence | metastatic hepatocellular carcinoma |
| models, cardiovascular | memory | metastatic her2-negative breast carcinoma |
| monitoring, ambulatory | memory, short-term | metastatic lung cancer |
| myocardial bridging | mental competency | metastatic lung carcinoma |
| myocardial contraction | mental disorders | metastatic lung non-small cell carcinoma |
| myocardial infarction | mental disorders diagnosed in childhood | metastatic lung non-squamous non-small cell carcinoma |
| myocardial injury | mental health | metastatic lung small cell carcinoma |
| myocardial ischemia | mental health services | metastatic malignant female reproductive system neoplasm |
| myocardial perfusion imaging | mental processes | metastatic malignant neoplasm |
| myocardial reperfusion | mental recall | metastatic malignant neoplasm in the bone |
| myocardial reperfusion injury | mental status schedule | metastatic malignant neoplasm in the brain |
| myocardial revascularization | methamphetamine addiction | metastatic malignant neoplasm in the central nervous system |
| myocardial stunning | methamphetamine dependence | metastatic malignant neoplasm in the liver |
| myocarditis | mild alzheimer's disease | metastatic malignant neoplasm in the lung |
| neovascularization, physiologic | mild cognitive impairment | metastatic malignant solid neoplasm |
| no-reflow phenomenon | mild traumatic brain injury | metastatic microsatellite stable colorectal carcinoma |
| orthostatic hypotension | milieu therapy | metastatic mucosal melanoma |
| pacemaker, artificial | military psychiatry | metastatic non small cell lung cancer |
| parasystole | minimal hepatic encephalopathy | metastatic non-small cell lung cancer |
| paroxysmal atrial fibrillation | mood | metastatic ovarian carcinoma |
| patent ductus arteriosus | mood disorder | metastatic pancreatic adenocarcinoma |
| percutaneous coronary intervention | mood disorders | metastatic pancreatic carcinoma |
| perfusion imaging | moral development | metastatic pancreatic ductal adenocarcinoma |
| pericardial effusion | mother-child relations | metastatic prostate adenocarcinoma |
| pericardiectomy | mothers | metastatic prostate carcinoma |
| pericarditis | motivation | metastatic rectal adenocarcinoma |
| pericarditis, constrictive | multiple personality disorder | metastatic renal pelvis urothelial carcinoma |
| pericarditis, tuberculous | nail biting | metastatic sarcoma |
| peripheral arterial disease | narcotherapy | metastatic soft tissue sarcoma |
| peripheral arterial disease (pad) | neurasthenia | metastatic solid tumor |
| peripheral arterial occlusive disease | neurocirculatory asthenia | metastatic triple negative breast cancer |
| peripheral artery disease | neuropathic pain | metastatic triple-negative breast carcinoma |
| peripheral vascular disease | neuropsychological tests | metastatic ureter urothelial carcinoma |
| peripheral vascular diseases | neuropsychology | metastatic urethral urothelial carcinoma |
| persistent atrial fibrillation | neurotic disorders | metastatic urothelial cancer |
| phonocardiography | nicotine dependence | metastatic urothelial carcinoma |
| pneumopericardium | nondirective therapy | metastatic uveal melanoma |
| postpericardiotomy syndrome | obsessive behavior | microsatellite stable colorectal cancer |
| postural tachycardia syndrome | obsessive compulsive disorder | mixed phenotype acute leukemia |
| pre-excitation syndromes | obsessive-compulsive disorder | mucosal melanoma |
| pre-excitation, mahaim-type | opiate addiction | multiple myeloma in relapse |
| pre-hypertension | opiate dependence | muscle invasive bladder cancer |
| primary hypercholesterolemia | opioid abuse | muscle invasive bladder carcinoma |
| pulmonary arterial hypertension | opioid dependence | myelodysplastic syndrome with excess blasts |
| pulmonary atresia | opioid dependency | myelodysplastic syndromes (mds) |
| pulmonary heart disease | oppositional defiant disorder | myelodysplastic/myeloproliferative neoplasm |
| pulmonary hypertension | optical illusions | myeloid leukemia |
| pulmonary subvalvular stenosis | orthopsychiatry | myeloid malignancy |
| pulmonary valve insufficiency | overlearning | myeloid neoplasm |
| pulmonary valve stenosis | pain management | myeloma multiple |
| pulmonary wedge pressure | pain relief | myeloproliferative disorder |
| pulse | paired-associate learning | myeloproliferative neoplasm |
| radionuclide angiography | panic | myeloproliferative neoplasms |
| radionuclide ventriculography | panic disorder | myxofibrosarcoma |
| reflex, oculocardiac | paranoid behavior | neoplasm malignant |
| regional blood flow | paranoid disorders | neuroendocrine carcinoma |
| renal artery stenosis | paranoid personality disorder | neuroendocrine neoplasm |
| reperfusion | paraphilias | newly diagnosed glioblastoma |
| reperfusion injury | parent-child relations | non small cell lung cancer metastatic |
| resistant hypertension | parents | non-melanoma skin cancer |
| restenosis | passive-aggressive personality disorder | non-muscle invasive bladder cancer |
| rheumatic heart disease | pastoral care | non-muscle invasive bladder neoplasms |
| romano-ward syndrome | paternal behavior | non-muscle-invasive bladder cancer |
| scimitar syndrome | paternal deprivation | non-small cell carcinoma |
| shock, cardiogenic | pathological gambling | non-small cell lung cancer metastatic |
| sick sinus syndrome | pattern recognition, physiological | non-small cell lung carcinoma |
| sinoatrial block | pedophilia | nonsmall cell lung cancer |
| sinus arrest, cardiac | perception | nonsmall cell lung cancer stage iii |
| situs inversus | perceptual distortion | nsclc stage iv |
| st-elevation myocardial infarction | personality | nsclc, recurrent |
| stable angina | personality disorders | nutrition aspect of cancer |
| stable coronary artery disease | phobic disorders | obesity-related malignant neoplasm |
| stent thrombosis | post traumatic stress disorder | ocular melanoma |
| stents | post-traumatic stress disorder | oligoprogressive |
| stroke prevention | post-traumatic stress disorders | oral cavity carcinoma |
| stroke volume | postpartum depression | oral cavity squamous cell carcinoma |
| stroke, acute | posttraumatic stress disorder | oropharyngeal carcinoma |
| sudden cardiac death | posttraumatic stress disorder (ptsd) | oropharyngeal squamous cell carcinoma |
| superior vena cava syndrome | posttraumatic stress disorders | oropharynx cancer |
| syphilis, cardiovascular | practice (psychology) | oropharynx squamous cell carcinoma |
| systole | premature ejaculation | other cancer |
| systolic murmurs | preventive psychiatry | other solid tumors |
| tachycardia | primary insomnia | ovarian carcinosarcoma |
| tachycardia, atrioventricular nodal reentry | proactive inhibition | ovarian clear cell adenocarcinoma |
| tachycardia, ectopic atrial | probability learning | ovarian clear cell carcinoma |
| tachycardia, ectopic junctional | problem solving | ovarian endometrioid adenocarcinoma |
| tachycardia, paroxysmal | professional misconduct | ovarian high grade serous adenocarcinoma |
| tachycardia, reciprocating | psychiatric somatic therapies | ovarian neoplasm |
| tachycardia, sinoatrial nodal reentry | psychiatric status rating scales | ovarian serous adenocarcinoma |
| tachycardia, sinus | psychiatry | ovary cancer |
| tachycardia, supraventricular | psychoanalysis | pancreas adenocarcinoma |
| tachycardia, ventricular | psychoanalytic theory | pancreatic cancer metastatic |
| takotsubo cardiomyopathy | psychoanalytic therapy | pancreatic cancer stage iv |
| telemetry | psychology, clinical | pancreatic carcinoma |
| tetralogy of fallot | psychology, experimental | pancreatic ductal adenocarcinoma |
| thrombolytic therapy | psychology, medical | pancreatic ductal adenocarcinoma (pdac) |
| tilt-table test | psychology, military | pancreatic neuroendocrine tumor |
| torsades de pointes | psychometrics | papillary renal cell carcinoma |
| transient ischemic attack | psychoneuroimmunology | papillary thyroid cancer |
| transposition of great vessels | psychopathology | parathyroid adenoma |
| tricuspid atresia | psychopharmacology | pathologic complete response |
| tricuspid valve insufficiency | psychosexual development | pathologic stage i hpv-mediated (p16-positive) oropharyngeal carcinoma ajcc v8 |
| tricuspid valve prolapse | psychosis | pathologic stage ic gastroesophageal junction adenocarcinoma ajcc v8 |
| tricuspid valve stenosis | psychosocial effects of cancer and its treatment | pathologic stage ii esophageal adenocarcinoma ajcc v8 |
| trilogy of fallot | psychosomatic medicine | pathologic stage ii gastroesophageal junction adenocarcinoma ajcc v8 |
| truncus arteriosus, persistent | psychotherapeutic processes | pathologic stage ii hpv-mediated (p16-positive) oropharyngeal carcinoma ajcc v8 |
| tuberculosis, cardiovascular | psychotherapy | pathologic stage iia esophageal adenocarcinoma ajcc v8 |
| unstable angina | psychotherapy, brief | pathologic stage iia gastroesophageal junction adenocarcinoma ajcc v8 |
| valve surgery | psychotherapy, group | pathologic stage iib esophageal adenocarcinoma ajcc v8 |
| vascular capacitance | psychotherapy, multiple | pathologic stage iib gastric cancer ajcc v8 |
| vascular disease | psychotherapy, rational-emotive | pathologic stage iib gastroesophageal junction adenocarcinoma ajcc v8 |
| vascular malformations | psychotic disorders | pathologic stage iii cutaneous melanoma ajcc v8 |
| vascular patency | ptsd | pathologic stage iii esophageal adenocarcinoma ajcc v8 |
| vascular resistance | punishment | pathologic stage iii gastric cancer ajcc v8 |
| vascular surgery | rage | pathologic stage iii gastroesophageal junction adenocarcinoma ajcc v8 |
| vascular surgical procedures | reactive attachment disorder | pathologic stage iii hpv-mediated (p16-positive) oropharyngeal carcinoma ajcc v8 |
| vasoconstriction | reactive inhibition | pathologic stage iiia cutaneous melanoma ajcc v8 |
| vasodilation | reality testing | pathologic stage iiia esophageal adenocarcinoma ajcc v8 |
| vectorcardiography | reality therapy | pathologic stage iiia gastric cancer ajcc v8 |
| venous pressure | recognition (psychology) | pathologic stage iiia gastroesophageal junction adenocarcinoma ajcc v8 |
| venous stasis ulcers | reinforcement (psychology) | pathologic stage iiib cutaneous melanoma ajcc v8 |
| ventricular arrythmias | reinforcement schedule | pathologic stage iiib esophageal adenocarcinoma ajcc v8 |
| ventricular dysfunction | reinforcement, social | pathologic stage iiib gastric cancer ajcc v8 |
| ventricular dysfunction, left | reinforcement, verbal | pathologic stage iiib gastroesophageal junction adenocarcinoma ajcc v8 |
| ventricular dysfunction, right | religion and psychology | pathologic stage iiic cutaneous melanoma ajcc v8 |
| ventricular fibrillation | residential treatment | pathologic stage iiic gastric cancer ajcc v8 |
| ventricular flutter | retention (psychology) | pathologic stage iiid cutaneous melanoma ajcc v8 |
| ventricular function | reversal learning | pathologic stage iv cutaneous melanoma ajcc v8 |
| ventricular function, left | reward | pathologic stage iv esophageal adenocarcinoma ajcc v8 |
| ventricular function, right | risk-taking | pathologic stage iv gastric cancer ajcc v8 |
| ventricular outflow obstruction | sadism | pathologic stage iv gastroesophageal junction adenocarcinoma ajcc v8 |
| ventricular premature complexes | safe sex | pathologic stage iva esophageal adenocarcinoma ajcc v8 |
| ventricular pressure | satiation | pathologic stage iva esophageal squamous cell carcinoma ajcc v8 |
| ventricular remodeling | satiety response | pathologic stage iva gastroesophageal junction adenocarcinoma ajcc v8 |
| ventricular septal rupture | schizoaffective | pathologic stage ivb esophageal adenocarcinoma ajcc v8 |
| ventricular tachycardia | schizoaffective disorder | pathologic stage ivb gastroesophageal junction adenocarcinoma ajcc v8 |
| ventriculography, first-pass | schizoid personality disorder | pediatric all |
| wolff-parkinson-white syndrome | schizophrenia | pediatric brain tumor |
|  | schizophrenia and disorders with psychotic features | pediatric cancer |
|  | schizophrenia, catatonic | pediatric solid tumor |
|  | schizophrenia, childhood | penile cancer |
|  | schizophrenia, disorganized | peripheral t cell lymphoma |
|  | schizophrenia, paranoid | peritoneal carcinoma |
|  | schizophrenic disorders | peritoneal carcinomatosis |
|  | schizophrenic language | peritoneal mesothelioma |
|  | schizophrenic psychology | philadelphia chromosome positive |
|  | schizophreniform disorder | plasma cell leukemia |
|  | schizotypal personality disorder | plasma cell myeloma |
|  | seasonal affective disorder | platinum-resistant fallopian tube carcinoma |
|  | sedation | platinum-resistant ovarian cancer |
|  | self stimulation | platinum-resistant ovarian carcinoma |
|  | self-injurious behavior | platinum-resistant primary peritoneal carcinoma |
|  | sensory deprivation | pleural mesothelioma |
|  | separation anxiety disorder | poorly differentiated thyroid gland carcinoma |
|  | serial learning | post-essential thrombocythemia myelofibrosis |
|  | sex counseling | post-polycythemia vera myelofibrosis |
|  | sex education | postneoadjuvant therapy stage iii gastric cancer ajcc v8 |
|  | sexology | postneoadjuvant therapy stage iii gastroesophageal junction adenocarcinoma ajcc v8 |
|  | sexual and gender disorders | postneoadjuvant therapy stage iiia gastroesophageal junction adenocarcinoma ajcc v8 |
|  | sexual behavior | postneoadjuvant therapy stage iiib gastroesophageal junction adenocarcinoma ajcc v8 |
|  | sexual dysfunction | postneoadjuvant therapy stage iv gastric cancer ajcc v8 |
|  | sexual dysfunctions, psychological | postneoadjuvant therapy stage iv gastroesophageal junction adenocarcinoma ajcc v8 |
|  | shared paranoid disorder | postneoadjuvant therapy stage iva gastroesophageal junction adenocarcinoma ajcc v8 |
|  | shyness | postneoadjuvant therapy stage ivb gastroesophageal junction adenocarcinoma ajcc v8 |
|  | sibling relations | pr-positive breast cancer |
|  | sleep disorder | primary brain tumor |
|  | sleep phase chronotherapy | primary central nervous system lymphoma |
|  | smoking | primary mediastinal large b cell lymphoma |
|  | smoking cessation | primary mediastinal large b-cell lymphoma (pmbcl) |
|  | social adjustment | primary peritoneal high grade serous adenocarcinoma |
|  | social anxiety disorder | progesterone receptor negative |
|  | social behavior | prognostic stage 0 breast cancer ajcc v8 |
|  | social phobia | prognostic stage i breast cancer ajcc v8 |
|  | social work, psychiatric | prognostic stage ia breast cancer ajcc v8 |
|  | socioenvironmental therapy | prognostic stage ib breast cancer ajcc v8 |
|  | somatoform disorders | prognostic stage ii breast cancer ajcc v8 |
|  | spirituality | prognostic stage iia breast cancer ajcc v8 |
|  | spouses | prognostic stage iib breast cancer ajcc v8 |
|  | stereotypic movement disorder | prognostic stage iii breast cancer ajcc v8 |
|  | stress | prognostic stage iiia breast cancer ajcc v8 |
|  | stress disorders, post-traumatic | prognostic stage iiib breast cancer ajcc v8 |
|  | stress disorders, traumatic | prognostic stage iiic breast cancer ajcc v8 |
|  | stress disorders, traumatic, acute | prognostic stage iv breast cancer ajcc v8 |
|  | stress, psychological | prostate adenocarcinoma |
|  | substance abuse | prostate cancer metastatic |
|  | substance dependence | prostate cancer recurrent |
|  | substance use | prostate carcinoma |
|  | substance use disorders | prostate carcinoma metastatic in the bone |
|  | suicide | prostate neoplasm |
|  | suicide, attempted | prostate neoplasms |
|  | temperament | prostatic cancer |
|  | test anxiety scale | prostatic neoplasm |
|  | theory of mind | rectal adenocarcinoma |
|  | therapeutic community | rectum cancer |
|  | thinking | recurrent acute biphenotypic leukemia |
|  | tobacco dependence | recurrent acute lymphoblastic leukemia |
|  | tobacco smoking | recurrent acute myeloid leukemia |
|  | tobacco use | recurrent b acute lymphoblastic leukemia |
|  | tobacco use cessation | recurrent b-cell non-hodgkin lymphoma |
|  | token economy | recurrent bladder urothelial carcinoma |
|  | transactional analysis | recurrent breast carcinoma |
|  | transference (psychology) | recurrent cancer |
|  | transient insomnia | recurrent cervical carcinoma |
|  | transsexualism | recurrent chronic lymphocytic leukemia |
|  | transvestism | recurrent chronic myelogenous leukemia, bcr-abl1 positive |
|  | treatment refusal | recurrent chronic myelomonocytic leukemia |
|  | treatment resistant depression | recurrent diffuse intrinsic pontine glioma |
|  | trichotillomania | recurrent diffuse large b-cell lymphoma |
|  | unipolar depression | recurrent diffuse large b-cell lymphoma, not otherwise specified |
|  | unsafe sex | recurrent endometrial cancer |
|  | verbal learning | recurrent endometrial carcinoma |
|  | voyeurism | recurrent ependymoma |
|  |  | recurrent ewing sarcoma |
|  |  | recurrent fallopian tube carcinoma |
|  |  | recurrent follicular lymphoma |
|  |  | recurrent glioma |
|  |  | recurrent gliosarcoma |
|  |  | recurrent head and neck cancer |
|  |  | recurrent head and neck carcinoma |
|  |  | recurrent head and neck squamous cell carcinoma |
|  |  | recurrent hepatoblastoma |
|  |  | recurrent hepatocellular carcinoma |
|  |  | recurrent high grade b-cell lymphoma |
|  |  | recurrent high grade b-cell lymphoma with myc and bcl2 or bcl6 rearrangements |
|  |  | recurrent high grade b-cell lymphoma with myc, bcl2, and bcl6 rearrangements |
|  |  | recurrent hodgkin lymphoma |
|  |  | recurrent hypopharyngeal squamous cell carcinoma |
|  |  | recurrent langerhans cell histiocytosis |
|  |  | recurrent laryngeal squamous cell carcinoma |
|  |  | recurrent lung non-small cell carcinoma |
|  |  | recurrent lung non-squamous non-small cell carcinoma |
|  |  | recurrent lung small cell carcinoma |
|  |  | recurrent lymphoma |
|  |  | recurrent malignant female reproductive system neoplasm |
|  |  | recurrent malignant germ cell tumor |
|  |  | recurrent malignant glioma |
|  |  | recurrent malignant solid neoplasm |
|  |  | recurrent medulloblastoma |
|  |  | recurrent mixed phenotype acute leukemia |
|  |  | recurrent multiple myeloma |
|  |  | recurrent mycosis fungoides |
|  |  | recurrent myelodysplastic syndrome |
|  |  | recurrent neuroblastoma |
|  |  | recurrent non-hodgkin lymphoma |
|  |  | recurrent oral cavity squamous cell carcinoma |
|  |  | recurrent oropharyngeal squamous cell carcinoma |
|  |  | recurrent osteosarcoma |
|  |  | recurrent ovarian cancer |
|  |  | recurrent ovarian carcinoma |
|  |  | recurrent ovarian endometrioid adenocarcinoma |
|  |  | recurrent ovarian high grade serous adenocarcinoma |
|  |  | recurrent peripheral primitive neuroectodermal tumor |
|  |  | recurrent plasma cell myeloma |
|  |  | recurrent platinum-resistant ovarian carcinoma |
|  |  | recurrent primary central nervous system neoplasm |
|  |  | recurrent primary cutaneous t-cell non-hodgkin lymphoma |
|  |  | recurrent primary mediastinal (thymic) large b-cell lymphoma |
|  |  | recurrent primary peritoneal carcinoma |
|  |  | recurrent prostate carcinoma |
|  |  | recurrent rhabdoid tumor |
|  |  | recurrent rhabdomyosarcoma |
|  |  | recurrent soft tissue sarcoma |
|  |  | recurrent who grade ii glioma |
|  |  | recurrent who grade iii glioma |
|  |  | refractory acute lymphoblastic leukemia |
|  |  | refractory acute myeloid leukemia |
|  |  | refractory b acute lymphoblastic leukemia |
|  |  | refractory b-cell non-hodgkin lymphoma |
|  |  | refractory breast carcinoma |
|  |  | refractory cancer |
|  |  | refractory chronic myelogenous leukemia, bcr-abl1 positive |
|  |  | refractory chronic myelomonocytic leukemia |
|  |  | refractory colorectal carcinoma |
|  |  | refractory diffuse large b-cell lymphoma |
|  |  | refractory diffuse large b-cell lymphoma, not otherwise specified |
|  |  | refractory ependymoma |
|  |  | refractory ewing sarcoma |
|  |  | refractory fallopian tube carcinoma |
|  |  | refractory follicular lymphoma |
|  |  | refractory hepatoblastoma |
|  |  | refractory high grade b-cell lymphoma |
|  |  | refractory high grade b-cell lymphoma with myc and bcl2 or bcl6 rearrangements |
|  |  | refractory high grade b-cell lymphoma with myc, bcl2, and bcl6 rearrangements |
|  |  | refractory hodgkin lymphoma |
|  |  | refractory langerhans cell histiocytosis |
|  |  | refractory lung non-small cell carcinoma |
|  |  | refractory lymphoma |
|  |  | refractory malignant germ cell tumor |
|  |  | refractory malignant glioma |
|  |  | refractory malignant solid neoplasm |
|  |  | refractory mantle cell lymphoma |
|  |  | refractory marginal zone lymphoma |
|  |  | refractory mature t-cell and nk-cell non-hodgkin lymphoma |
|  |  | refractory medulloblastoma |
|  |  | refractory mixed phenotype acute leukemia |
|  |  | refractory mycosis fungoides |
|  |  | refractory myelodysplastic syndrome |
|  |  | refractory neuroblastoma |
|  |  | refractory non-hodgkin lymphoma |
|  |  | refractory osteosarcoma |
|  |  | refractory ovarian carcinoma |
|  |  | refractory peripheral primitive neuroectodermal tumor |
|  |  | refractory plasma cell myeloma |
|  |  | refractory primary central nervous system neoplasm |
|  |  | refractory primary cutaneous t-cell non-hodgkin lymphoma |
|  |  | refractory primary mediastinal (thymic) large b-cell lymphoma |
|  |  | refractory primary peritoneal carcinoma |
|  |  | refractory rhabdoid tumor |
|  |  | refractory rhabdomyosarcoma |
|  |  | refractory small lymphocytic lymphoma |
|  |  | refractory soft tissue sarcoma |
|  |  | refractory transformed follicular lymphoma to diffuse large b-cell lymphoma |
|  |  | relapse multiple myeloma |
|  |  | relapsed acute myeloid leukemia |
|  |  | relapsed adult aml |
|  |  | relapsed cancer |
|  |  | relapsed diffuse large b-cell lymphoma |
|  |  | relapsed neuroblastoma |
|  |  | relapsed non hodgkin lymphoma |
|  |  | relapsed non-hodgkin lymphoma |
|  |  | relapsed or refractory acute myeloid leukemia |
|  |  | relapsed/refractory follicular lymphoma |
|  |  | relapsed/refractory multiple myeloma |
|  |  | renal cell carcinoma (rcc) |
|  |  | resectable hepatocellular carcinoma |
|  |  | resectable pancreatic ductal adenocarcinoma |
|  |  | richter syndrome |
|  |  | salivary gland cancer |
|  |  | sarcoma,soft tissue |
|  |  | sarcomatoid renal cell carcinoma |
|  |  | sclc |
|  |  | skin carcinoma |
|  |  | small cell lung cancer (sclc) |
|  |  | small lymphocytic lymphoma (sll) |
|  |  | small-cell lung cancer |
|  |  | smoldering plasma cell myeloma |
|  |  | solid carcinoma |
|  |  | solid tumor, adult |
|  |  | solid tumor, unspecified, adult |
|  |  | solid tumors, adult |
|  |  | spine metastases |
|  |  | squamous cell carcinoma of the head and neck (scchn) |
|  |  | squamous cell carcinoma of the oropharynx |
|  |  | stage 0a bladder cancer ajcc v8 |
|  |  | stage 0is bladder cancer ajcc v8 |
|  |  | stage i bladder cancer ajcc v8 |
|  |  | stage i breast cancer ajcc v7 |
|  |  | stage i colorectal cancer ajcc v8 |
|  |  | stage i lung cancer ajcc v8 |
|  |  | stage i pancreatic cancer ajcc v8 |
|  |  | stage i prostate cancer ajcc v8 |
|  |  | stage ia breast cancer ajcc v7 |
|  |  | stage ia1 lung cancer ajcc v8 |
|  |  | stage ia2 lung cancer ajcc v8 |
|  |  | stage ia3 lung cancer ajcc v8 |
|  |  | stage ib breast cancer ajcc v7 |
|  |  | stage ib lung cancer ajcc v8 |
|  |  | stage ii bladder cancer ajcc v8 |
|  |  | stage ii breast cancer ajcc v6 and v7 |
|  |  | stage ii colorectal cancer ajcc v8 |
|  |  | stage ii lung cancer ajcc v8 |
|  |  | stage ii pancreatic cancer ajcc v8 |
|  |  | stage ii prostate cancer ajcc v8 |
|  |  | stage ii rectal cancer ajcc v8 |
|  |  | stage iia breast cancer ajcc v6 and v7 |
|  |  | stage iia colorectal cancer ajcc v8 |
|  |  | stage iia lung cancer ajcc v8 |
|  |  | stage iia pancreatic cancer ajcc v8 |
|  |  | stage iia prostate cancer ajcc v8 |
|  |  | stage iib breast cancer ajcc v6 and v7 |
|  |  | stage iib colorectal cancer ajcc v8 |
|  |  | stage iib lung cancer ajcc v8 |
|  |  | stage iib pancreatic cancer ajcc v8 |
|  |  | stage iib prostate cancer ajcc v8 |
|  |  | stage iic colorectal cancer ajcc v8 |
|  |  | stage iic prostate cancer ajcc v8 |
|  |  | stage iii bladder cancer ajcc v8 |
|  |  | stage iii breast cancer |
|  |  | stage iii breast cancer ajcc v7 |
|  |  | stage iii cervical cancer ajcc v8 |
|  |  | stage iii colon cancer ajcc v8 |
|  |  | stage iii colorectal cancer |
|  |  | stage iii colorectal cancer ajcc v8 |
|  |  | stage iii cutaneous squamous cell carcinoma of the head and neck ajcc v8 |
|  |  | stage iii fallopian tube cancer ajcc v8 |
|  |  | stage iii gallbladder cancer ajcc v8 |
|  |  | stage iii hepatocellular carcinoma ajcc v8 |
|  |  | stage iii hypopharyngeal carcinoma ajcc v8 |
|  |  | stage iii intrahepatic cholangiocarcinoma ajcc v8 |
|  |  | stage iii laryngeal cancer ajcc v8 |
|  |  | stage iii lip and oral cavity cancer ajcc v8 |
|  |  | stage iii lung cancer ajcc v8 |
|  |  | stage iii oropharyngeal (p16-negative) carcinoma ajcc v8 |
|  |  | stage iii ovarian cancer ajcc v8 |
|  |  | stage iii pancreatic cancer |
|  |  | stage iii pancreatic cancer ajcc v8 |
|  |  | stage iii primary peritoneal cancer ajcc v8 |
|  |  | stage iii prostate cancer ajcc v8 |
|  |  | stage iii rectal cancer ajcc v8 |
|  |  | stage iii renal cell cancer ajcc v8 |
|  |  | stage iii renal pelvis cancer ajcc v8 |
|  |  | stage iii soft tissue sarcoma of the trunk and extremities ajcc v8 |
|  |  | stage iii ureter cancer ajcc v8 |
|  |  | stage iii urethral cancer ajcc v8 |
|  |  | stage iii uterine corpus cancer ajcc v8 |
|  |  | stage iiia bladder cancer ajcc v8 |
|  |  | stage iiia breast cancer ajcc v7 |
|  |  | stage iiia colon cancer ajcc v8 |
|  |  | stage iiia colorectal cancer ajcc v8 |
|  |  | stage iiia fallopian tube cancer ajcc v8 |
|  |  | stage iiia hepatocellular carcinoma ajcc v8 |
|  |  | stage iiia intrahepatic cholangiocarcinoma ajcc v8 |
|  |  | stage iiia lung cancer ajcc v8 |
|  |  | stage iiia ovarian cancer ajcc v8 |
|  |  | stage iiia primary peritoneal cancer ajcc v8 |
|  |  | stage iiia prostate cancer ajcc v8 |
|  |  | stage iiia rectal cancer ajcc v8 |
|  |  | stage iiia uterine corpus cancer ajcc v8 |
|  |  | stage iiia1 fallopian tube cancer ajcc v8 |
|  |  | stage iiia1 ovarian cancer ajcc v8 |
|  |  | stage iiia2 fallopian tube cancer ajcc v8 |
|  |  | stage iiia2 ovarian cancer ajcc v8 |
|  |  | stage iiib bladder cancer ajcc v8 |
|  |  | stage iiib breast cancer ajcc v7 |
|  |  | stage iiib cervical cancer ajcc v8 |
|  |  | stage iiib colon cancer ajcc v8 |
|  |  | stage iiib colorectal cancer ajcc v8 |
|  |  | stage iiib fallopian tube cancer ajcc v8 |
|  |  | stage iiib hepatocellular carcinoma ajcc v8 |
|  |  | stage iiib intrahepatic cholangiocarcinoma ajcc v8 |
|  |  | stage iiib lung cancer ajcc v8 |
|  |  | stage iiib ovarian cancer ajcc v8 |
|  |  | stage iiib primary peritoneal cancer ajcc v8 |
|  |  | stage iiib prostate cancer ajcc v8 |
|  |  | stage iiib rectal cancer ajcc v8 |
|  |  | stage iiib uterine corpus cancer ajcc v8 |
|  |  | stage iiic breast cancer ajcc v7 |
|  |  | stage iiic colon cancer ajcc v8 |
|  |  | stage iiic colorectal cancer ajcc v8 |
|  |  | stage iiic fallopian tube cancer ajcc v8 |
|  |  | stage iiic lung cancer ajcc v8 |
|  |  | stage iiic ovarian cancer ajcc v8 |
|  |  | stage iiic primary peritoneal cancer ajcc v8 |
|  |  | stage iiic prostate cancer ajcc v8 |
|  |  | stage iiic rectal cancer ajcc v8 |
|  |  | stage iiic uterine corpus cancer ajcc v8 |
|  |  | stage iiic1 uterine corpus cancer ajcc v8 |
|  |  | stage iiic2 uterine corpus cancer ajcc v8 |
|  |  | stage iv bladder cancer ajcc v8 |
|  |  | stage iv breast cancer ajcc v6 and v7 |
|  |  | stage iv cervical cancer ajcc v8 |
|  |  | stage iv colon cancer ajcc v8 |
|  |  | stage iv colorectal cancer |
|  |  | stage iv colorectal cancer ajcc v7 |
|  |  | stage iv colorectal cancer ajcc v8 |
|  |  | stage iv cutaneous squamous cell carcinoma of the head and neck ajcc v8 |
|  |  | stage iv distal bile duct cancer ajcc v8 |
|  |  | stage iv fallopian tube cancer ajcc v8 |
|  |  | stage iv hepatocellular carcinoma ajcc v8 |
|  |  | stage iv hypopharyngeal carcinoma ajcc v8 |
|  |  | stage iv intrahepatic cholangiocarcinoma ajcc v8 |
|  |  | stage iv laryngeal cancer ajcc v8 |
|  |  | stage iv lip and oral cavity cancer ajcc v8 |
|  |  | stage iv lung cancer |
|  |  | stage iv lung cancer ajcc v8 |
|  |  | stage iv major salivary gland cancer ajcc v8 |
|  |  | stage iv non-small cell lung cancer |
|  |  | stage iv oropharyngeal (p16-negative) carcinoma ajcc v8 |
|  |  | stage iv ovarian cancer ajcc v8 |
|  |  | stage iv pancreatic cancer |
|  |  | stage iv pancreatic cancer ajcc v8 |
|  |  | stage iv primary peritoneal cancer ajcc v8 |
|  |  | stage iv prostate adenocarcinoma ajcc v7 |
|  |  | stage iv prostate cancer ajcc v8 |
|  |  | stage iv rectal cancer ajcc v8 |
|  |  | stage iv renal cell cancer ajcc v8 |
|  |  | stage iv renal pelvis cancer ajcc v8 |
|  |  | stage iv ureter cancer ajcc v8 |
|  |  | stage iv urethral cancer ajcc v8 |
|  |  | stage iv uterine corpus cancer ajcc v8 |
|  |  | stage iva bladder cancer ajcc v8 |
|  |  | stage iva cervical cancer ajcc v8 |
|  |  | stage iva colon cancer ajcc v8 |
|  |  | stage iva colorectal cancer ajcc v7 |
|  |  | stage iva colorectal cancer ajcc v8 |
|  |  | stage iva fallopian tube cancer ajcc v8 |
|  |  | stage iva hepatocellular carcinoma ajcc v8 |
|  |  | stage iva hypopharyngeal carcinoma ajcc v8 |
|  |  | stage iva laryngeal cancer ajcc v8 |
|  |  | stage iva lip and oral cavity cancer ajcc v8 |
|  |  | stage iva lung cancer ajcc v8 |
|  |  | stage iva oropharyngeal (p16-negative) carcinoma ajcc v8 |
|  |  | stage iva ovarian cancer ajcc v8 |
|  |  | stage iva primary peritoneal cancer ajcc v8 |
|  |  | stage iva prostate cancer ajcc v8 |
|  |  | stage iva rectal cancer ajcc v8 |
|  |  | stage iva uterine corpus cancer ajcc v8 |
|  |  | stage iva vaginal cancer ajcc v8 |
|  |  | stage ivb bladder cancer ajcc v8 |
|  |  | stage ivb cervical cancer ajcc v8 |
|  |  | stage ivb colon cancer ajcc v8 |
|  |  | stage ivb colorectal cancer ajcc v7 |
|  |  | stage ivb colorectal cancer ajcc v8 |
|  |  | stage ivb fallopian tube cancer ajcc v8 |
|  |  | stage ivb hepatocellular carcinoma ajcc v8 |
|  |  | stage ivb hypopharyngeal carcinoma ajcc v8 |
|  |  | stage ivb laryngeal cancer ajcc v8 |
|  |  | stage ivb lip and oral cavity cancer ajcc v8 |
|  |  | stage ivb lung cancer ajcc v8 |
|  |  | stage ivb oropharyngeal (p16-negative) carcinoma ajcc v8 |
|  |  | stage ivb ovarian cancer ajcc v8 |
|  |  | stage ivb primary peritoneal cancer ajcc v8 |
|  |  | stage ivb prostate cancer ajcc v8 |
|  |  | stage ivb rectal cancer ajcc v8 |
|  |  | stage ivb uterine corpus cancer ajcc v8 |
|  |  | stage ivc colon cancer ajcc v8 |
|  |  | stage ivc colorectal cancer ajcc v8 |
|  |  | stage ivc rectal cancer ajcc v8 |
|  |  | superficial basal cell carcinoma |
|  |  | synovial sarcoma |
|  |  | t acute lymphoblastic leukemia |
|  |  | t-cell lymphoma |
|  |  | t-cell/histiocyte-rich large b-cell lymphoma |
|  |  | tenosynovial giant cell tumor |
|  |  | therapy-related acute myeloid leukemia |
|  |  | therapy-related myelodysplastic syndrome |
|  |  | thoracic cancer |
|  |  | thyroid gland carcinoma |
|  |  | tnbc - triple-negative breast cancer |
|  |  | tobacco-related carcinoma |
|  |  | transformed follicular lymphoma to diffuse large b-cell lymphoma |
|  |  | transformed lymphoma |
|  |  | triple negative breast cancer (tnbc) |
|  |  | triple-negative breast cancer |
|  |  | triple-negative breast carcinoma |
|  |  | tumor, solid |
|  |  | undifferentiated pleomorphic sarcoma |
|  |  | unresectable breast carcinoma |
|  |  | unresectable colon adenocarcinoma |
|  |  | unresectable colorectal carcinoma |
|  |  | unresectable gastroesophageal junction adenocarcinoma |
|  |  | unresectable head and neck squamous cell carcinoma |
|  |  | unresectable hepatocellular carcinoma |
|  |  | unresectable intrahepatic cholangiocarcinoma |
|  |  | unresectable lung non-small cell carcinoma |
|  |  | unresectable malignant solid neoplasm |
|  |  | unresectable melanoma |
|  |  | unresectable pancreatic adenocarcinoma |
|  |  | unresectable pancreatic carcinoma |
|  |  | unresectable rectal adenocarcinoma |
|  |  | unresectable renal cell carcinoma |
|  |  | unresectable soft tissue sarcoma |
|  |  | unresectable solid neoplasm |
|  |  | unresectable urothelial carcinoma |
|  |  | urologic cancer |
|  |  | urothelial carcinoma |
|  |  | urothelial carcinoma bladder |
|  |  | who grade iii glioma |
|  |  | abdominal neoplasms |
|  |  | adenocarcinoma |
|  |  | adenocarcinoma, bronchiolo-alveolar |
|  |  | adenocarcinoma, clear cell |
|  |  | adenocarcinoma, follicular |
|  |  | adenocarcinoma, mucinous |
|  |  | adenocarcinoma, papillary |
|  |  | adenocarcinoma, scirrhous |
|  |  | adenocarcinoma, sebaceous |
|  |  | adenosarcoma |
|  |  | adrenal cortex neoplasms |
|  |  | adrenal gland neoplasms |
|  |  | adrenocortical carcinoma |
|  |  | anal gland neoplasms |
|  |  | anus neoplasms |
|  |  | appendiceal neoplasms |
|  |  | bile duct neoplasms |
|  |  | biliary tract neoplasms |
|  |  | bone marrow neoplasms |
|  |  | bone neoplasms |
|  |  | brain neoplasms |
|  |  | brain stem neoplasms |
|  |  | brain tumor |
|  |  | breast neoplasms |
|  |  | breast neoplasms, male |
|  |  | bronchial neoplasms |
|  |  | carcinoid syndrome |
|  |  | carcinoid tumor |
|  |  | carcinoma |
|  |  | carcinoma 256, walker |
|  |  | carcinoma in situ |
|  |  | carcinoma, acinar cell |
|  |  | carcinoma, adenoid cystic |
|  |  | carcinoma, adenosquamous |
|  |  | carcinoma, basal cell |
|  |  | carcinoma, basosquamous |
|  |  | carcinoma, bronchogenic |
|  |  | carcinoma, brown-pearce |
|  |  | carcinoma, ductal |
|  |  | carcinoma, ductal, breast |
|  |  | carcinoma, ehrlich tumor |
|  |  | carcinoma, embryonal |
|  |  | carcinoma, endometrioid |
|  |  | carcinoma, giant cell |
|  |  | carcinoma, hepatocellular |
|  |  | carcinoma, intraductal, noninfiltrating |
|  |  | carcinoma, islet cell |
|  |  | carcinoma, krebs 2 |
|  |  | carcinoma, large cell |
|  |  | carcinoma, lewis lung |
|  |  | carcinoma, lobular |
|  |  | carcinoma, medullary |
|  |  | carcinoma, merkel cell |
|  |  | carcinoma, mucoepidermoid |
|  |  | carcinoma, neuroendocrine |
|  |  | carcinoma, non-small-cell lung |
|  |  | carcinoma, pancreatic ductal |
|  |  | carcinoma, papillary |
|  |  | carcinoma, papillary, follicular |
|  |  | carcinoma, renal cell |
|  |  | carcinoma, signet ring cell |
|  |  | carcinoma, skin appendage |
|  |  | carcinoma, small cell |
|  |  | carcinoma, squamous cell |
|  |  | carcinoma, transitional cell |
|  |  | carcinoma, verrucous |
|  |  | carcinosarcoma |
|  |  | cecal neoplasms |
|  |  | central nervous system neoplasms |
|  |  | cerebellar neoplasms |
|  |  | cerebral ventricle neoplasms |
|  |  | cholangiocarcinoma |
|  |  | chondrosarcoma |
|  |  | chondrosarcoma, mesenchymal |
|  |  | choriocarcinoma |
|  |  | choriocarcinoma, non-gestational |
|  |  | choroid neoplasms |
|  |  | choroid plexus neoplasms |
|  |  | colonic neoplasms |
|  |  | colorectal neoplasms |
|  |  | colorectal neoplasms, hereditary nonpolyposis |
|  |  | common bile duct neoplasms |
|  |  | conjunctival neoplasms |
|  |  | cranial nerve neoplasms |
|  |  | cystadenocarcinoma |
|  |  | cystadenocarcinoma, mucinous |
|  |  | cystadenocarcinoma, papillary |
|  |  | cystadenocarcinoma, serous |
|  |  | dendritic cell sarcoma, follicular |
|  |  | dendritic cell sarcoma, interdigitating |
|  |  | dermatofibrosarcoma |
|  |  | digestive system neoplasms |
|  |  | drug resistance, neoplasm |
|  |  | duodenal neoplasms |
|  |  | ear neoplasms |
|  |  | eccrine porocarcinoma |
|  |  | endocrine gland neoplasms |
|  |  | endodermal sinus tumor |
|  |  | endometrial neoplasms |
|  |  | endometrial stromal tumors |
|  |  | epidural neoplasms |
|  |  | esophageal neoplasms |
|  |  | eye neoplasms |
|  |  | eyelid neoplasms |
|  |  | facial neoplasms |
|  |  | fallopian tube neoplasms |
|  |  | femoral neoplasms |
|  |  | fibrosarcoma |
|  |  | gallbladder neoplasms |
|  |  | gastrointestinal neoplasms |
|  |  | gastrointestinal stromal tumors |
|  |  | genes, neoplasm |
|  |  | genital neoplasms, female |
|  |  | genital neoplasms, male |
|  |  | gingival neoplasms |
|  |  | gliosarcoma |
|  |  | granulosa cell tumor |
|  |  | head and neck neoplasms |
|  |  | heart neoplasms |
|  |  | hemangiosarcoma |
|  |  | hematologic neoplasms |
|  |  | histiocytic sarcoma |
|  |  | hypopharyngeal neoplasms |
|  |  | hypothalamic neoplasms |
|  |  | ileal neoplasms |
|  |  | infratentorial neoplasms |
|  |  | intestinal neoplasms |
|  |  | iris neoplasms |
|  |  | jaw neoplasms |
|  |  | jejunal neoplasms |
|  |  | kidney neoplasms |
|  |  | klatskin's tumor |
|  |  | krukenberg tumor |
|  |  | langerhans cell sarcoma |
|  |  | laryngeal neoplasms |
|  |  | leiomyosarcoma |
|  |  | lip neoplasms |
|  |  | liposarcoma |
|  |  | liposarcoma, myxoid |
|  |  | liver neoplasms |
|  |  | liver neoplasms, experimental |
|  |  | lung neoplasms |
|  |  | lymphangiosarcoma |
|  |  | mammary neoplasms, animal |
|  |  | mammary neoplasms, experimental |
|  |  | mandibular neoplasms |
|  |  | mast-cell sarcoma |
|  |  | maxillary neoplasms |
|  |  | maxillary sinus neoplasms |
|  |  | mediastinal neoplasms |
|  |  | meningeal carcinomatosis |
|  |  | meningeal neoplasms |
|  |  | mixed tumor, malignant |
|  |  | mixed tumor, mesodermal |
|  |  | mixed tumor, mullerian |
|  |  | mouth neoplasms |
|  |  | mucoepidermoid tumor |
|  |  | muscle neoplasms |
|  |  | myosarcoma |
|  |  | myxosarcoma |
|  |  | nasopharyngeal neoplasms |
|  |  | neoplasm invasiveness |
|  |  | neoplasm metastasis |
|  |  | neoplasm recurrence, local |
|  |  | neoplasm regression, spontaneous |
|  |  | neoplasm seeding |
|  |  | neoplasm staging |
|  |  | neoplasm transplantation |
|  |  | neoplasm, residual |
|  |  | neoplasms |
|  |  | neoplasms by histologic type |
|  |  | neoplasms by site |
|  |  | neoplasms, adipose tissue |
|  |  | neoplasms, adnexal and skin appendage |
|  |  | neoplasms, basal cell |
|  |  | neoplasms, bone tissue |
|  |  | neoplasms, complex and mixed |
|  |  | neoplasms, connective and soft tissue |
|  |  | neoplasms, connective tissue |
|  |  | neoplasms, cystic, mucinous, and serous |
|  |  | neoplasms, ductal, lobular, and medullary |
|  |  | neoplasms, experimental |
|  |  | neoplasms, fibroepithelial |
|  |  | neoplasms, fibrous tissue |
|  |  | neoplasms, germ cell and embryonal |
|  |  | neoplasms, glandular and epithelial |
|  |  | neoplasms, gonadal tissue |
|  |  | neoplasms, hormone-dependent |
|  |  | neoplasms, mesothelial |
|  |  | neoplasms, multiple primary |
|  |  | neoplasms, muscle tissue |
|  |  | neoplasms, nerve tissue |
|  |  | neoplasms, neuroepithelial |
|  |  | neoplasms, plasma cell |
|  |  | neoplasms, post-traumatic |
|  |  | neoplasms, radiation-induced |
|  |  | neoplasms, second primary |
|  |  | neoplasms, squamous cell |
|  |  | neoplasms, unknown primary |
|  |  | neoplasms, vascular tissue |
|  |  | neoplastic cells, circulating |
|  |  | neoplastic processes |
|  |  | neoplastic syndromes, hereditary |
|  |  | nerve sheath neoplasms |
|  |  | nervous system neoplasms |
|  |  | neuroectodermal tumor, melanotic |
|  |  | neuroectodermal tumors |
|  |  | neuroectodermal tumors, primitive |
|  |  | neuroectodermal tumors, primitive, peripheral |
|  |  | neuroendocrine tumors |
|  |  | neurofibrosarcoma |
|  |  | nose neoplasms |
|  |  | optic nerve neoplasms |
|  |  | orbital neoplasms |
|  |  | oropharyngeal neoplasms |
|  |  | osteosarcoma |
|  |  | osteosarcoma, juxtacortical |
|  |  | otorhinolaryngologic neoplasms |
|  |  | ovarian neoplasms |
|  |  | palatal neoplasms |
|  |  | pancreatic neoplasms |
|  |  | paranasal sinus neoplasms |
|  |  | parathyroid neoplasms |
|  |  | parotid neoplasms |
|  |  | pelvic neoplasms |
|  |  | penile neoplasms |
|  |  | peripheral nervous system neoplasms |
|  |  | peritoneal neoplasms |
|  |  | perivascular epithelioid cell neoplasms |
|  |  | pharyngeal neoplasms |
|  |  | pituitary neoplasms |
|  |  | pleural neoplasms |
|  |  | prostatic neoplasms |
|  |  | rectal neoplasms |
|  |  | respiratory tract neoplasms |
|  |  | retinal neoplasms |
|  |  | retroperitoneal neoplasms |
|  |  | rhabdoid tumor |
|  |  | rhabdomyosarcoma |
|  |  | rhabdomyosarcoma, alveolar |
|  |  | rhabdomyosarcoma, embryonal |
|  |  | salivary gland neoplasms |
|  |  | sarcoma |
|  |  | sarcoma 180 |
|  |  | sarcoma 37 |
|  |  | sarcoma, alveolar soft part |
|  |  | sarcoma, avian |
|  |  | sarcoma, clear cell |
|  |  | sarcoma, endometrial stromal |
|  |  | sarcoma, ewing |
|  |  | sarcoma, experimental |
|  |  | sarcoma, kaposi |
|  |  | sarcoma, myeloid |
|  |  | sarcoma, small cell |
|  |  | sarcoma, synovial |
|  |  | sarcoma, yoshida |
|  |  | sebaceous gland neoplasms |
|  |  | sigmoid neoplasms |
|  |  | skin neoplasms |
|  |  | skull base neoplasms |
|  |  | skull neoplasms |
|  |  | small cell lung carcinoma |
|  |  | soft tissue neoplasms |
|  |  | spinal cord neoplasms |
|  |  | spinal neoplasms |
|  |  | splenic neoplasms |
|  |  | stomach neoplasms |
|  |  | sublingual gland neoplasms |
|  |  | submandibular gland neoplasms |
|  |  | supratentorial neoplasms |
|  |  | sweat gland neoplasms |
|  |  | teratocarcinoma |
|  |  | testicular neoplasms |
|  |  | thoracic neoplasms |
|  |  | thymus neoplasms |
|  |  | thyroid neoplasms |
|  |  | tongue neoplasms |
|  |  | tonsillar neoplasms |
|  |  | tracheal neoplasms |
|  |  | trophoblastic neoplasms |
|  |  | ureteral neoplasms |
|  |  | urethral neoplasms |
|  |  | urinary bladder neoplasms |
|  |  | urogenital neoplasms |
|  |  | urologic neoplasms |
|  |  | uterine cervical neoplasms |
|  |  | uterine neoplasms |
|  |  | uveal neoplasms |
|  |  | vaginal neoplasms |
|  |  | vascular neoplasms |
|  |  | vulvar neoplasms |
|  |  | waldenstrom macroglobulinemia |
|  |  | wilms tumor |
|  |  | adenocarcinoma of the gastroesophageal junction |
|  |  | adenocarcinoma of the prostate |
|  |  | ewing's sarcoma |
|  |  | †abdominal neoplasms |
|  |  | †acanthoma |
|  |  | †acth syndrome, ectopic |
|  |  | †adamantinoma |
|  |  | †adenocarcinoma |
|  |  | †adenocarcinoma, bronchiolo-alveolar |
|  |  | †adenocarcinoma, clear cell |
|  |  | †adenocarcinoma, follicular |
|  |  | †adenocarcinoma, mucinous |
|  |  | †adenocarcinoma, papillary |
|  |  | †adenocarcinoma, scirrhous |
|  |  | †adenocarcinoma, sebaceous |
|  |  | †adenomatous polyposis coli |
|  |  | †adenomatous polyps |
|  |  | †adenosarcoma |
|  |  | †adrenal cortex neoplasms |
|  |  | †adrenal gland neoplasms |
|  |  | †adrenocortical carcinoma |
|  |  | †ameloblastoma |
|  |  | †anus neoplasms |
|  |  | †appendiceal neoplasms |
|  |  | †astrocytoma |
|  |  | †bile duct neoplasms |
|  |  | †biliary tract neoplasms |
|  |  | †bone neoplasms |
|  |  | †bowen's disease |
|  |  | †brain neoplasms |
|  |  | †brain stem neoplasms |
|  |  | †breast neoplasms |
|  |  | †breast neoplasms, male |
|  |  | †bronchial neoplasms |
|  |  | †burkitt lymphoma |
|  |  | †carcinoid tumor |
|  |  | †carcinoma |
|  |  | †carcinoma in situ |
|  |  | †carcinoma, acinar cell |
|  |  | †carcinoma, adenoid cystic |
|  |  | †carcinoma, adenosquamous |
|  |  | †carcinoma, basal cell |
|  |  | †carcinoma, basosquamous |
|  |  | †carcinoma, bronchogenic |
|  |  | †carcinoma, ductal |
|  |  | †carcinoma, ductal, breast |
|  |  | †carcinoma, embryonal |
|  |  | †carcinoma, endometrioid |
|  |  | †carcinoma, giant cell |
|  |  | †carcinoma, hepatocellular |
|  |  | †carcinoma, intraductal, noninfiltrating |
|  |  | †carcinoma, islet cell |
|  |  | †carcinoma, large cell |
|  |  | †carcinoma, lobular |
|  |  | †carcinoma, medullary |
|  |  | †carcinoma, merkel cell |
|  |  | †carcinoma, mucoepidermoid |
|  |  | †carcinoma, neuroendocrine |
|  |  | †carcinoma, non-small-cell lung |
|  |  | †carcinoma, pancreatic ductal |
|  |  | †carcinoma, papillary |
|  |  | †carcinoma, papillary, follicular |
|  |  | †carcinoma, renal cell |
|  |  | †carcinoma, signet ring cell |
|  |  | †carcinoma, skin appendage |
|  |  | †carcinoma, small cell |
|  |  | †carcinoma, squamous cell |
|  |  | †carcinoma, transitional cell |
|  |  | †carcinoma, verrucous |
|  |  | †carotid body tumor |
|  |  | †cecal neoplasms |
|  |  | †central nervous system neoplasms |
|  |  | †cerebellar neoplasms |
|  |  | †cervical intraepithelial neoplasia |
|  |  | †cholangiocarcinoma |
|  |  | †chondroblastoma |
|  |  | †chondrosarcoma |
|  |  | †chondrosarcoma, mesenchymal |
|  |  | †chordoma |
|  |  | †choriocarcinoma |
|  |  | †choriocarcinoma, non-gestational |
|  |  | †choroid neoplasms |
|  |  | †colonic neoplasms |
|  |  | †colorectal neoplasms |
|  |  | †common bile duct neoplasms |
|  |  | †conjunctival neoplasms |
|  |  | †cranial nerve neoplasms |
|  |  | †craniopharyngioma |
|  |  | †cystadenocarcinoma |
|  |  | †cystadenocarcinoma, mucinous |
|  |  | †cystadenocarcinoma, papillary |
|  |  | †cystadenocarcinoma, serous |
|  |  | †cystadenoma |
|  |  | †cystadenoma, mucinous |
|  |  | †cystadenoma, papillary |
|  |  | †cystadenoma, serous |
|  |  | †dermatofibrosarcoma |
|  |  | †digestive system neoplasms |
|  |  | †duodenal neoplasms |
|  |  | †dysgerminoma |
|  |  | †dysplastic nevus syndrome |
|  |  | †ear neoplasms |
|  |  | †eccrine porocarcinoma |
|  |  | †endocrine gland neoplasms |
|  |  | †endodermal sinus tumor |
|  |  | †endometrial neoplasms |
|  |  | †endometrial stromal tumors |
|  |  | †ependymoma |
|  |  | †epidural neoplasms |
|  |  | †erythroplasia |
|  |  | †esophageal neoplasms |
|  |  | †eye neoplasms |
|  |  | †eyelid neoplasms |
|  |  | †facial neoplasms |
|  |  | †fallopian tube neoplasms |
|  |  | †femoral neoplasms |
|  |  | †fibrosarcoma |
|  |  | †gallbladder neoplasms |
|  |  | †ganglioglioma |
|  |  | †ganglioneuroblastoma |
|  |  | †ganglioneuroma |
|  |  | †gastrinoma |
|  |  | †gastrointestinal neoplasms |
|  |  | †gastrointestinal stromal tumors |
|  |  | †genes, abl |
|  |  | †genes, apc |
|  |  | †genes, bcl-1 |
|  |  | †genes, bcl-2 |
|  |  | †genes, brca1 |
|  |  | †genes, brca2 |
|  |  | †genes, dcc |
|  |  | †genes, erba |
|  |  | †genes, erbb |
|  |  | †genes, erbb-1 |
|  |  | †genes, erbb-2 |
|  |  | †genes, fms |
|  |  | †genes, fos |
|  |  | †genes, jun |
|  |  | †genes, mcc |
|  |  | †genes, mos |
|  |  | †genes, myb |
|  |  | †genes, myc |
|  |  | †genes, neoplasm |
|  |  | †genes, neurofibromatosis 1 |
|  |  | †genes, neurofibromatosis 2 |
|  |  | †genes, p16 |
|  |  | †genes, p53 |
|  |  | †genes, ras |
|  |  | †genes, rel |
|  |  | †genes, retinoblastoma |
|  |  | †genes, sis |
|  |  | †genes, tumor suppressor |
|  |  | †genes, wilms tumor |
|  |  | †genital neoplasms, female |
|  |  | †genital neoplasms, male |
|  |  | †germinoma |
|  |  | †gestational trophoblastic neoplasms |
|  |  | †giant cell tumor of bone |
|  |  | †giant cell tumors |
|  |  | †gingival neoplasms |
|  |  | †glioblastoma |
|  |  | †glioma |
|  |  | †glioma, subependymal |
|  |  | †gliosarcoma |
|  |  | †glomus jugulare tumor |
|  |  | †glomus tympanicum tumor |
|  |  | †glucagonoma |
|  |  | †gonadoblastoma |
|  |  | †granular cell tumor |
|  |  | †granulosa cell tumor |
|  |  | †head and neck neoplasms |
|  |  | †heart neoplasms |
|  |  | †hemangioblastoma |
|  |  | †hemangioendothelioma |
|  |  | †hemangioendothelioma, epithelioid |
|  |  | †hemangiosarcoma |
|  |  | †hematologic neoplasms |
|  |  | †hepatoblastoma |
|  |  | †histiocytoma, malignant fibrous |
|  |  | †hodgkin disease |
|  |  | †hutchinson's melanotic freckle |
|  |  | †hypopharyngeal neoplasms |
|  |  | †hypothalamic neoplasms |
|  |  | †ileal neoplasms |
|  |  | †immunoproliferative disorders |
|  |  | †infratentorial neoplasms |
|  |  | †intestinal neoplasms |
|  |  | †iris neoplasms |
|  |  | †jaw neoplasms |
|  |  | †jejunal neoplasms |
|  |  | †kidney neoplasms |
|  |  | †klatskin's tumor |
|  |  | †krukenberg tumor |
|  |  | †laryngeal neoplasms |
|  |  | †leiomyosarcoma |
|  |  | †leukemia-lymphoma, adult t-cell |
|  |  | †leukemia, b-cell |
|  |  | †leukemia, basophilic, acute |
|  |  | †leukemia, biphenotypic, acute |
|  |  | †leukemia, eosinophilic, acute |
|  |  | †leukemia, erythroblastic, acute |
|  |  | †leukemia, hairy cell |
|  |  | †leukemia, large granular lymphocytic |
|  |  | †leukemia, lymphocytic, chronic, b-cell |
|  |  | †leukemia, lymphoid |
|  |  | †leukemia, mast-cell |
|  |  | †leukemia, megakaryoblastic, acute |
|  |  | †leukemia, monocytic, acute |
|  |  | †leukemia, myelogenous, chronic, bcr-abl positive |
|  |  | †leukemia, myeloid |
|  |  | †leukemia, myeloid, accelerated phase |
|  |  | †leukemia, myeloid, acute |
|  |  | †leukemia, myeloid, chronic-phase |
|  |  | †leukemia, myeloid, chronic, atypical, bcr-abl negative |
|  |  | †leukemia, myelomonocytic, acute |
|  |  | †leukemia, myelomonocytic, chronic |
|  |  | †leukemia, myelomonocytic, juvenile |
|  |  | †leukemia, neutrophilic, chronic |
|  |  | †leukemia, plasma cell |
|  |  | †leukemia, prolymphocytic |
|  |  | †leukemia, prolymphocytic, b-cell |
|  |  | †leukemia, prolymphocytic, t-cell |
|  |  | †leukemia, promyelocytic, acute |
|  |  | †leukemia, t-cell |
|  |  | †leydig cell tumor |
|  |  | †linitis plastica |
|  |  | †lip neoplasms |
|  |  | †liposarcoma |
|  |  | †liposarcoma, myxoid |
|  |  | †liver neoplasms |
|  |  | †lung neoplasms |
|  |  | †lymphangiosarcoma |
|  |  | †lymphatic vessel tumors |
|  |  | †lymphoma |
|  |  | †lymphoma, aids-related |
|  |  | †lymphoma, b-cell |
|  |  | †lymphoma, b-cell, marginal zone |
|  |  | †lymphoma, extranodal nk-t-cell |
|  |  | †lymphoma, follicular |
|  |  | †lymphoma, large b-cell, diffuse |
|  |  | †lymphoma, large-cell, anaplastic |
|  |  | †lymphoma, large-cell, immunoblastic |
|  |  | †lymphoma, mantle-cell |
|  |  | †lymphoma, non-hodgkin |
|  |  | †lymphoma, primary cutaneous anaplastic large cell |
|  |  | †lymphoma, primary effusion |
|  |  | †lymphoma, t-cell |
|  |  | †lymphoma, t-cell, cutaneous |
|  |  | †lymphoma, t-cell, peripheral |
|  |  | †lymphomatoid granulomatosis |
|  |  | †lymphomatoid papulosis |
|  |  | †lynch syndrome ii |
|  |  | †malignant carcinoid syndrome |
|  |  | †mandibular neoplasms |
|  |  | †mast-cell sarcoma |
|  |  | †mastocytosis, systemic |
|  |  | †maxillary neoplasms |
|  |  | †maxillary sinus neoplasms |
|  |  | †mediastinal neoplasms |
|  |  | †medulloblastoma |
|  |  | †melanoma |
|  |  | †melanoma, amelanotic |
|  |  | †meningeal carcinomatosis |
|  |  | †meningeal neoplasms |
|  |  | †meningioma |
|  |  | †mesenchymoma |
|  |  | †mesonephroma |
|  |  | †mesothelioma |
|  |  | †mesothelioma, cystic |
|  |  | †mixed tumor, malignant |
|  |  | †mixed tumor, mesodermal |
|  |  | †mixed tumor, mullerian |
|  |  | †mouth neoplasms |
|  |  | †mucoepidermoid tumor |
|  |  | †multiple endocrine neoplasia |
|  |  | †multiple endocrine neoplasia type 1 |
|  |  | †multiple endocrine neoplasia type 2a |
|  |  | †multiple endocrine neoplasia type 2b |
|  |  | †multiple myeloma |
|  |  | †multiple pulmonary nodules |
|  |  | †muscle neoplasms |
|  |  | †mycosis fungoides |
|  |  | †myelodysplastic syndromes |
|  |  | †myeloproliferative disorders |
|  |  | †myoepithelioma |
|  |  | †myosarcoma |
|  |  | †myxoma |
|  |  | †myxosarcoma |
|  |  | †nasopharyngeal neoplasms |
|  |  | †neoplasms |
|  |  | †neoplasms by histologic type |
|  |  | †neoplasms by site |
|  |  | †neoplasms, adnexal and skin appendage |
|  |  | †neoplasms, basal cell |
|  |  | †neoplasms, bone tissue |
|  |  | †neoplasms, complex and mixed |
|  |  | †neoplasms, connective and soft tissue |
|  |  | †neoplasms, cystic, mucinous, and serous |
|  |  | †neoplasms, ductal, lobular, and medullary |
|  |  | †neoplasms, fibrous tissue |
|  |  | †neoplasms, germ cell and embryonal |
|  |  | †neoplasms, glandular and epithelial |
|  |  | †neoplasms, gonadal tissue |
|  |  | †neoplasms, hormone-dependent |
|  |  | †neoplasms, mesothelial |
|  |  | †neoplasms, multiple primary |
|  |  | †neoplasms, muscle tissue |
|  |  | †neoplasms, nerve tissue |
|  |  | †neoplasms, neuroepithelial |
|  |  | †neoplasms, plasma cell |
|  |  | †neoplasms, post-traumatic |
|  |  | †neoplasms, second primary |
|  |  | †neoplasms, squamous cell |
|  |  | †neoplasms, vascular tissue |
|  |  | †neoplastic syndromes, hereditary |
|  |  | †nephroma, mesoblastic |
|  |  | †nerve sheath neoplasms |
|  |  | †nervous system neoplasms |
|  |  | †neurilemmoma |
|  |  | †neuroblastoma |
|  |  | †neuroectodermal tumor, melanotic |
|  |  | †neuroectodermal tumors |
|  |  | †neuroectodermal tumors, primitive |
|  |  | †neuroectodermal tumors, primitive, peripheral |
|  |  | †neuroendocrine tumors |
|  |  | †neurofibroma |
|  |  | †neurofibroma, plexiform |
|  |  | †neurofibromatoses |
|  |  | †neurofibromatosis 1 |
|  |  | †neurofibromatosis 2 |
|  |  | †neurofibrosarcoma |
|  |  | †neuroma |
|  |  | †neuroma, acoustic |
|  |  | †nevi and melanomas |
|  |  | †nose neoplasms |
|  |  | †odontogenic tumors |
|  |  | †oligodendroglioma |
|  |  | †oncogenes |
|  |  | †optic nerve glioma |
|  |  | †optic nerve neoplasms |
|  |  | †orbital neoplasms |
|  |  | †oropharyngeal neoplasms |
|  |  | †osteoblastoma |
|  |  | †osteosarcoma |
|  |  | †osteosarcoma, juxtacortical |
|  |  | †otorhinolaryngologic neoplasms |
|  |  | †ovarian neoplasms |
|  |  | †paget disease extramammary |
|  |  | †paget's disease, mammary |
|  |  | †pagetoid reticulosis |
|  |  | †palatal neoplasms |
|  |  | †pancoast syndrome |
|  |  | †pancreatic neoplasms |
|  |  | †paraganglioma |
|  |  | †paraganglioma, extra-adrenal |
|  |  | †paranasal sinus diseases |
|  |  | †paranasal sinus neoplasms |
|  |  | †paraneoplastic cerebellar degeneration |
|  |  | †paraneoplastic endocrine syndromes |
|  |  | †paraneoplastic polyneuropathy |
|  |  | †paraneoplastic syndromes |
|  |  | †paraneoplastic syndromes, nervous system |
|  |  | †parathyroid neoplasms |
|  |  | †parotid neoplasms |
|  |  | †pelvic neoplasms |
|  |  | †penile neoplasms |
|  |  | †peripheral nervous system neoplasms |
|  |  | †pharyngeal neoplasms |
|  |  | †pheochromocytoma |
|  |  | †phyllodes tumor |
|  |  | †plasmacytoma |
|  |  | †pleural effusion |
|  |  | †pleural effusion, malignant |
|  |  | †pleural neoplasms |
|  |  | †precancerous conditions |
|  |  | †precursor b-cell lymphoblastic leukemia-lymphoma |
|  |  | †precursor cell lymphoblastic leukemia-lymphoma |
|  |  | †precursor t-cell lymphoblastic leukemia-lymphoma |
|  |  | †preleukemia |
|  |  | †prostatic intraepithelial neoplasia |
|  |  | †prostatic neoplasms |
|  |  | †proto-oncogenes |
|  |  | †pseudomyxoma peritonei |
|  |  | †pulmonary blastoma |
|  |  | †rectal neoplasms |
|  |  | †respiratory tract neoplasms |
|  |  | †retinal neoplasms |
|  |  | †retinoblastoma |
|  |  | †retroperitoneal neoplasms |
|  |  | †rhabdoid tumor |
|  |  | †rhabdomyosarcoma |
|  |  | †rhabdomyosarcoma, alveolar |
|  |  | †rhabdomyosarcoma, embryonal |
|  |  | †salivary gland neoplasms |
|  |  | †sarcoma |
|  |  | †sarcoma, alveolar soft part |
|  |  | †sarcoma, clear cell |
|  |  | †sarcoma, endometrial stromal |
|  |  | †sarcoma, ewing's |
|  |  | †sarcoma, kaposi |
|  |  | †sarcoma, myeloid |
|  |  | †sarcoma, small cell |
|  |  | †sarcoma, synovial |
|  |  | †schnitzler syndrome |
|  |  | †seminoma |
|  |  | †sertoli cell tumor |
|  |  | †sertoli-leydig cell tumor |
|  |  | †sex cord-gonadal stromal tumors |
|  |  | †sigmoid neoplasms |
|  |  | †skin neoplasms |
|  |  | †skull base neoplasms |
|  |  | †skull neoplasms |
|  |  | †small cell lung carcinoma |
|  |  | †soft tissue neoplasms |
|  |  | †solitary pulmonary nodule |
|  |  | †somatostatinoma |
|  |  | †spinal cord neoplasms |
|  |  | †splenic neoplasms |
|  |  | †stomach neoplasms |
|  |  | †struma ovarii |
|  |  | †sublingual gland neoplasms |
|  |  | †submandibular gland neoplasms |
|  |  | †supratentorial neoplasms |
|  |  | †sweat gland neoplasms |
|  |  | †teratocarcinoma |
|  |  | †teratoma |
|  |  | †testicular neoplasms |
|  |  | †thoracic neoplasms |
|  |  | †thymoma |
|  |  | †thymus neoplasms |
|  |  | †tongue neoplasms |
|  |  | †tonsillar neoplasms |
|  |  | †tracheal neoplasms |
|  |  | †trophoblastic tumor, placental site |
|  |  | †ureteral neoplasms |
|  |  | †urethral neoplasms |
|  |  | †urinary bladder neoplasms |
|  |  | †urogenital neoplasms |
|  |  | †urologic neoplasms |
|  |  | †uterine cervical neoplasms |
|  |  | †uterine neoplasms |
|  |  | †uveal neoplasms |
|  |  | †vaginal neoplasms |
|  |  | †vascular neoplasms |
|  |  | †vipoma |
|  |  | †vulvar neoplasms |
|  |  | †wilms tumor |
|  |  | †zollinger-ellison syndrome |
|  |  | accelerated phase chronic myelogenous leukemia |
|  |  | acute leukemia |
|  |  | acute lymphoblastic leukemia |
|  |  | acute lymphocytic leukemia |
|  |  | acute myelogenous leukemia |
|  |  | acute myeloid leukemia |
|  |  | acute myeloid leukemia (aml) |
|  |  | acute promyelocytic leukemia |
|  |  | adult acute lymphoblastic leukemia in remission |
|  |  | adult acute megakaryoblastic leukemia (m7) |
|  |  | adult acute monoblastic leukemia (m5a) |
|  |  | adult acute monocytic leukemia (m5b) |
|  |  | adult acute myeloid leukemia in remission |
|  |  | adult acute myeloid leukemia with 11q23 (mll) abnormalities |
|  |  | adult acute myeloid leukemia with del(5q) |
|  |  | adult acute myeloid leukemia with inv(16)(p13;q22) |
|  |  | adult acute myeloid leukemia with t(15;17)(q22;q12) |
|  |  | adult acute myeloid leukemia with t(16;16)(p13;q22) |
|  |  | adult acute myeloid leukemia with t(8;21)(q22;q22) |
|  |  | adult anaplastic astrocytoma |
|  |  | adult anaplastic oligodendroglioma |
|  |  | adult erythroleukemia (m6a) |
|  |  | adult giant cell glioblastoma |
|  |  | adult glioblastoma |
|  |  | adult gliosarcoma |
|  |  | adult mixed glioma |
|  |  | adult nasal type extranodal nk/t-cell lymphoma |
|  |  | adult pure erythroid leukemia (m6b) |
|  |  | advanced breast cancer |
|  |  | advanced cancer |
|  |  | advanced cancers |
|  |  | advanced colorectal cancer |
|  |  | advanced gastric cancer |
|  |  | advanced hepatocellular carcinoma |
|  |  | advanced malignancies |
|  |  | advanced malignant solid tumors |
|  |  | advanced melanoma |
|  |  | advanced non-small cell lung cancer |
|  |  | advanced or metastatic solid tumors |
|  |  | advanced renal cell carcinoma |
|  |  | advanced solid malignancies |
|  |  | advanced solid tumor |
|  |  | advanced solid tumors |
|  |  | advanced solid tumours |
|  |  | all |
|  |  | allogeneic stem cell transplantation |
|  |  | aml |
|  |  | anal cancer |
|  |  | anaplastic astrocytoma |
|  |  | anaplastic large cell lymphoma |
|  |  | anaplastic oligodendroglioma |
|  |  | angioimmunoblastic t-cell lymphoma |
|  |  | b-cell chronic lymphocytic leukemia |
|  |  | b-cell lymphoma |
|  |  | basal cell carcinoma |
|  |  | bile duct cancer |
|  |  | biliary cancer |
|  |  | biliary tract cancer |
|  |  | bladder cancer |
|  |  | blastic phase chronic myelogenous leukemia |
|  |  | bone metastases |
|  |  | brain and central nervous system tumors |
|  |  | brain cancer |
|  |  | brain metastases |
|  |  | brain tumors |
|  |  | breast cancer |
|  |  | breast carcinoma |
|  |  | breast neoplasm |
|  |  | breast tumors |
|  |  | cancer |
|  |  | cancer of the breast |
|  |  | cancer of the prostate |
|  |  | cancer survivor |
|  |  | carcinoma, non small cell lung |
|  |  | carcinoma, non-small cell lung |
|  |  | central nervous system tumors |
|  |  | cervical cancer |
|  |  | cervical neoplasia |
|  |  | chemotherapeutic agent toxicity |
|  |  | chemotherapy |
|  |  | chemotherapy-induced nausea and vomiting |
|  |  | childhood acute lymphoblastic leukemia in remission |
|  |  | childhood acute myeloid leukemia in remission |
|  |  | childhood chronic myelogenous leukemia |
|  |  | childhood germ cell tumor |
|  |  | childhood myelodysplastic syndromes |
|  |  | chronic eosinophilic leukemia |
|  |  | chronic lymphocytic leukemia |
|  |  | chronic lymphocytic leukemia (cll) |
|  |  | chronic myelogenous leukemia |
|  |  | chronic myeloid leukemia |
|  |  | chronic myelomonocytic leukemia |
|  |  | chronic myeloproliferative disorders |
|  |  | chronic neutrophilic leukemia |
|  |  | chronic phase chronic myelogenous leukemia |
|  |  | clear cell renal cell carcinoma |
|  |  | cll |
|  |  | cml |
|  |  | cns cancer |
|  |  | colon cancer |
|  |  | colorectal cancer |
|  |  | colorectal cancer screening |
|  |  | colorectal carcinoma |
|  |  | colorectal neoplasm |
|  |  | contiguous stage ii adult burkitt lymphoma |
|  |  | contiguous stage ii adult diffuse large cell lymphoma |
|  |  | contiguous stage ii adult diffuse mixed cell lymphoma |
|  |  | contiguous stage ii adult diffuse small cleaved cell lymphoma |
|  |  | contiguous stage ii adult immunoblastic large cell lymphoma |
|  |  | contiguous stage ii adult lymphoblastic lymphoma |
|  |  | contiguous stage ii grade 1 follicular lymphoma |
|  |  | contiguous stage ii grade 2 follicular lymphoma |
|  |  | contiguous stage ii grade 3 follicular lymphoma |
|  |  | contiguous stage ii mantle cell lymphoma |
|  |  | contiguous stage ii marginal zone lymphoma |
|  |  | contiguous stage ii small lymphocytic lymphoma |
|  |  | cutaneous b-cell non-hodgkin lymphoma |
|  |  | cutaneous t-cell lymphoma |
|  |  | de novo myelodysplastic syndromes |
|  |  | diffuse large b-cell lymphoma |
|  |  | disease, hodgkin |
|  |  | ductal carcinoma in situ |
|  |  | endometrial cancer |
|  |  | epithelial ovarian cancer |
|  |  | esophageal cancer |
|  |  | esophageal carcinoma |
|  |  | extragonadal germ cell tumor |
|  |  | extrahepatic bile duct cancer |
|  |  | extranodal marginal zone b-cell lymphoma of mucosa-associated lymphoid tissue |
|  |  | fallopian tube cancer |
|  |  | familial adenomatous polyposis |
|  |  | follicular lymphoma |
|  |  | gallbladder cancer |
|  |  | gastric cancer |
|  |  | gastrointestinal cancer |
|  |  | gastrointestinal carcinoid tumor |
|  |  | gastrointestinal stromal tumor |
|  |  | gastrointestinal stromal tumor (gist) |
|  |  | gbm |
|  |  | glioblastoma multiforme |
|  |  | head & neck cancer |
|  |  | head and neck cancer |
|  |  | head and neck cancers |
|  |  | head and neck squamous cell carcinoma |
|  |  | hematologic malignancies |
|  |  | hematological malignancies |
|  |  | hematological malignancy |
|  |  | hematopoietic/lymphoid cancer |
|  |  | hepatocellular cancer |
|  |  | hepatocellular carcinoma |
|  |  | hepatoma |
|  |  | hepatosplenic t-cell lymphoma |
|  |  | hodgkin lymphoma |
|  |  | hodgkin's disease |
|  |  | hodgkin's lymphoma |
|  |  | hodgkins disease |
|  |  | hormone refractory prostate cancer |
|  |  | hormone-refractory prostate cancer |
|  |  | intraocular lymphoma |
|  |  | intraocular melanoma |
|  |  | invasive breast cancer |
|  |  | islet cell tumor |
|  |  | juvenile myelomonocytic leukemia |
|  |  | kidney cancer |
|  |  | leukemia, lymphoblastic, acute |
|  |  | leukemia, lymphocytic, acute |
|  |  | leukemia, lymphocytic, chronic |
|  |  | leukemia, myeloid, chronic |
|  |  | liver cancer |
|  |  | liver metastases |
|  |  | liver metastasis |
|  |  | liver tumors |
|  |  | localized prostate cancer |
|  |  | locally advanced pancreatic cancer |
|  |  | long-term effects secondary to cancer therapy in adults |
|  |  | lung cancer |
|  |  | lymphoblastic leukemia |
|  |  | lymphoblastic lymphoma |
|  |  | lymphocytic leukemia, chronic |
|  |  | lymphoma, small lymphocytic |
|  |  | lymphomas |
|  |  | lymphomas: non-hodgkin |
|  |  | lymphoproliferative disorder |
|  |  | male breast cancer |
|  |  | malignancy |
|  |  | malignant ascites |
|  |  | malignant glioma |
|  |  | malignant gliomas |
|  |  | malignant melanoma |
|  |  | malignant mesothelioma |
|  |  | malignant pleural mesothelioma |
|  |  | mantle cell lymphoma |
|  |  | mantle-cell lymphoma |
|  |  | marginal zone lymphoma |
|  |  | mds |
|  |  | melanoma (skin) |
|  |  | metastases |
|  |  | metastasis |
|  |  | metastatic breast cancer |
|  |  | metastatic cancer |
|  |  | metastatic colorectal cancer |
|  |  | metastatic melanoma |
|  |  | metastatic pancreatic cancer |
|  |  | metastatic prostate cancer |
|  |  | metastatic renal cell carcinoma |
|  |  | metastatic solid tumors |
|  |  | multiple myeloma and plasma cell neoplasm |
|  |  | myelodysplasia |
|  |  | myelodysplastic syndrome |
|  |  | myelodysplastic syndrome (mds) |
|  |  | myelodysplastic/myeloproliferative diseases |
|  |  | myelodysplastic/myeloproliferative neoplasm, unclassifiable |
|  |  | myelodysplastic/myeloproliferative neoplasms |
|  |  | myelofibrosis |
|  |  | myeloma |
|  |  | nasopharyngeal cancer |
|  |  | nasopharyngeal carcinoma |
|  |  | neoplasm |
|  |  | neoplasms, breast |
|  |  | neoplasms, colorectal |
|  |  | neoplasms, malignant |
|  |  | neuroendocrine tumor |
|  |  | nhl |
|  |  | nodal marginal zone b-cell lymphoma |
|  |  | non hodgkin lymphoma |
|  |  | non hodgkin's lymphoma |
|  |  | non small cell lung cancer |
|  |  | non small cell lung cancer (nsclc) |
|  |  | non small cell lung carcinoma |
|  |  | non-hodgkin lymphoma |
|  |  | non-hodgkin's lymphoma |
|  |  | non-hodgkin's lymphoma (nhl) |
|  |  | non-hodgkins lymphoma |
|  |  | non-melanomatous skin cancer |
|  |  | non-small cell lung cancer |
|  |  | non-small cell lung cancer (nsclc) |
|  |  | non-small-cell lung cancer |
|  |  | non-small-cell lung carcinoma |
|  |  | noncontiguous stage ii adult burkitt lymphoma |
|  |  | noncontiguous stage ii adult diffuse large cell lymphoma |
|  |  | noncontiguous stage ii adult diffuse mixed cell lymphoma |
|  |  | noncontiguous stage ii adult diffuse small cleaved cell lymphoma |
|  |  | noncontiguous stage ii adult immunoblastic large cell lymphoma |
|  |  | noncontiguous stage ii adult lymphoblastic lymphoma |
|  |  | noncontiguous stage ii grade 1 follicular lymphoma |
|  |  | noncontiguous stage ii grade 2 follicular lymphoma |
|  |  | noncontiguous stage ii grade 3 follicular lymphoma |
|  |  | noncontiguous stage ii mantle cell lymphoma |
|  |  | noncontiguous stage ii marginal zone lymphoma |
|  |  | noncontiguous stage ii small lymphocytic lymphoma |
|  |  | nonmalignant neoplasm |
|  |  | nsclc |
|  |  | oncology |
|  |  | oral cancer |
|  |  | oropharyngeal cancer |
|  |  | ovarian cancer |
|  |  | ovarian carcinoma |
|  |  | pancreas cancer |
|  |  | pancreatic adenocarcinoma |
|  |  | pancreatic cancer |
|  |  | pancreatic neoplasm |
|  |  | peripheral t-cell lymphoma |
|  |  | peritoneal cancer |
|  |  | peritoneal cavity cancer |
|  |  | post-transplant lymphoproliferative disorder |
|  |  | precancerous condition |
|  |  | previously treated myelodysplastic syndromes |
|  |  | primary breast cancer |
|  |  | primary peritoneal cancer |
|  |  | primary peritoneal carcinoma |
|  |  | prolymphocytic leukemia |
|  |  | prostate cancer |
|  |  | rectal cancer |
|  |  | recurrent adult acute lymphoblastic leukemia |
|  |  | recurrent adult acute myeloid leukemia |
|  |  | recurrent adult brain tumor |
|  |  | recurrent adult burkitt lymphoma |
|  |  | recurrent adult diffuse large cell lymphoma |
|  |  | recurrent adult diffuse mixed cell lymphoma |
|  |  | recurrent adult diffuse small cleaved cell lymphoma |
|  |  | recurrent adult hodgkin lymphoma |
|  |  | recurrent adult immunoblastic large cell lymphoma |
|  |  | recurrent adult lymphoblastic lymphoma |
|  |  | recurrent adult t-cell leukemia/lymphoma |
|  |  | recurrent breast cancer |
|  |  | recurrent childhood acute lymphoblastic leukemia |
|  |  | recurrent childhood acute myeloid leukemia |
|  |  | recurrent childhood large cell lymphoma |
|  |  | recurrent childhood lymphoblastic lymphoma |
|  |  | recurrent childhood small noncleaved cell lymphoma |
|  |  | recurrent colon cancer |
|  |  | recurrent cutaneous t-cell non-hodgkin lymphoma |
|  |  | recurrent glioblastoma |
|  |  | recurrent grade 1 follicular lymphoma |
|  |  | recurrent grade 2 follicular lymphoma |
|  |  | recurrent grade 3 follicular lymphoma |
|  |  | recurrent malignant testicular germ cell tumor |
|  |  | recurrent mantle cell lymphoma |
|  |  | recurrent marginal zone lymphoma |
|  |  | recurrent mycosis fungoides/sezary syndrome |
|  |  | recurrent ovarian epithelial cancer |
|  |  | recurrent ovarian germ cell tumor |
|  |  | recurrent prostate cancer |
|  |  | recurrent rectal cancer |
|  |  | recurrent small lymphocytic lymphoma |
|  |  | refractory anemia with excess blasts |
|  |  | refractory anemia with ringed sideroblasts |
|  |  | refractory chronic lymphocytic leukemia |
|  |  | refractory cytopenia with multilineage dysplasia |
|  |  | refractory hairy cell leukemia |
|  |  | refractory multiple myeloma |
|  |  | refractory solid tumors |
|  |  | relapsed multiple myeloma |
|  |  | relapsed or refractory multiple myeloma |
|  |  | relapsing chronic myelogenous leukemia |
|  |  | renal cancer |
|  |  | renal cell cancer |
|  |  | renal cell carcinoma |
|  |  | secondary acute myeloid leukemia |
|  |  | secondary myelodysplastic syndromes |
|  |  | secondary myelofibrosis |
|  |  | skin cancer |
|  |  | small cell lung cancer |
|  |  | small intestine cancer |
|  |  | small intestine lymphoma |
|  |  | small lymphocytic lymphoma |
|  |  | soft tissue sarcoma |
|  |  | solid cancers |
|  |  | solid malignancies |
|  |  | solid tumor |
|  |  | solid tumor cancer |
|  |  | solid tumors |
|  |  | splenic marginal zone lymphoma |
|  |  | squamous cell cancer |
|  |  | squamous cell carcinoma |
|  |  | squamous cell carcinoma of the head and neck |
|  |  | stage i adult burkitt lymphoma |
|  |  | stage i adult diffuse large cell lymphoma |
|  |  | stage i adult diffuse mixed cell lymphoma |
|  |  | stage i adult diffuse small cleaved cell lymphoma |
|  |  | stage i adult immunoblastic large cell lymphoma |
|  |  | stage i adult lymphoblastic lymphoma |
|  |  | stage i breast cancer |
|  |  | stage i chronic lymphocytic leukemia |
|  |  | stage i cutaneous t-cell non-hodgkin lymphoma |
|  |  | stage i grade 1 follicular lymphoma |
|  |  | stage i grade 2 follicular lymphoma |
|  |  | stage i grade 3 follicular lymphoma |
|  |  | stage i mantle cell lymphoma |
|  |  | stage i marginal zone lymphoma |
|  |  | stage i multiple myeloma |
|  |  | stage i prostate cancer |
|  |  | stage i small lymphocytic lymphoma |
|  |  | stage ii breast cancer |
|  |  | stage ii chronic lymphocytic leukemia |
|  |  | stage ii cutaneous t-cell non-hodgkin lymphoma |
|  |  | stage ii multiple myeloma |
|  |  | stage ii prostate cancer |
|  |  | stage iii adult burkitt lymphoma |
|  |  | stage iii adult diffuse large cell lymphoma |
|  |  | stage iii adult diffuse mixed cell lymphoma |
|  |  | stage iii adult diffuse small cleaved cell lymphoma |
|  |  | stage iii adult hodgkin lymphoma |
|  |  | stage iii adult immunoblastic large cell lymphoma |
|  |  | stage iii adult lymphoblastic lymphoma |
|  |  | stage iii chronic lymphocytic leukemia |
|  |  | stage iii cutaneous t-cell non-hodgkin lymphoma |
|  |  | stage iii grade 1 follicular lymphoma |
|  |  | stage iii grade 2 follicular lymphoma |
|  |  | stage iii grade 3 follicular lymphoma |
|  |  | stage iii mantle cell lymphoma |
|  |  | stage iii marginal zone lymphoma |
|  |  | stage iii multiple myeloma |
|  |  | stage iii mycosis fungoides/sezary syndrome |
|  |  | stage iii ovarian epithelial cancer |
|  |  | stage iii prostate cancer |
|  |  | stage iii small lymphocytic lymphoma |
|  |  | stage iiia breast cancer |
|  |  | stage iiib breast cancer |
|  |  | stage iiic breast cancer |
|  |  | stage iv adult burkitt lymphoma |
|  |  | stage iv adult diffuse large cell lymphoma |
|  |  | stage iv adult diffuse mixed cell lymphoma |
|  |  | stage iv adult diffuse small cleaved cell lymphoma |
|  |  | stage iv adult hodgkin lymphoma |
|  |  | stage iv adult immunoblastic large cell lymphoma |
|  |  | stage iv adult lymphoblastic lymphoma |
|  |  | stage iv breast cancer |
|  |  | stage iv chronic lymphocytic leukemia |
|  |  | stage iv colon cancer |
|  |  | stage iv cutaneous t-cell non-hodgkin lymphoma |
|  |  | stage iv grade 1 follicular lymphoma |
|  |  | stage iv grade 2 follicular lymphoma |
|  |  | stage iv grade 3 follicular lymphoma |
|  |  | stage iv mantle cell lymphoma |
|  |  | stage iv marginal zone lymphoma |
|  |  | stage iv melanoma |
|  |  | stage iv mycosis fungoides/sezary syndrome |
|  |  | stage iv ovarian epithelial cancer |
|  |  | stage iv prostate cancer |
|  |  | stage iv rectal cancer |
|  |  | stage iv renal cell cancer |
|  |  | stage iv small lymphocytic lymphoma |
|  |  | stomach cancer |
|  |  | testicular germ cell tumor |
|  |  | testicular lymphoma |
|  |  | thymic carcinoma |
|  |  | thyroid cancer |
|  |  | thyroid carcinoma |
|  |  | thyroid neoplasm |
|  |  | transitional cell cancer of the renal pelvis and ureter |
|  |  | transitional cell carcinoma |
|  |  | triple negative breast cancer |
|  |  | tumor |
|  |  | tumors |
|  |  | unspecified adult solid tumor, protocol specific |
|  |  | unspecified childhood solid tumor, protocol specific |
|  |  | untreated adult acute myeloid leukemia |
|  |  | urethral cancer |
|  |  | urothelial cancer |
|  |  | uterine cancer |
|  |  | uveal melanoma |
|  |  | vaginal cancer |
|  |  | vulvar cancer |
|  |  | waldenstrom's macroglobulinemia |

**Supplement 3: Data Sharing Statement**

The datasets analyzed during the current study are publicly available on the Clinical Trials Transformation Initiative website, <https://aact.ctti-clinicaltrials.org/>
